# Development of a CRISPR-Cas12a system for efficient genome engineering in clostridia

**DOI:** 10.1128/spectrum.02459-23

**Published:** 2023-11-10

**Authors:** Yanchao Zhang, Aleksandra M. Kubiak, Tom S. Bailey, Luuk Claessen, Philip Hittmeyer, Ludwig Dubois, Jan Theys, Philippe Lambin

**Affiliations:** 1 M-Lab, Department of Precision Medicine, GROW - School of Oncology and Reproduction, Maastricht University, Maastricht, The Netherlands; 2 Exomnis Biotech BV, Maastricht, The Netherlands; 3 LivingMed Biotech SRL, Liège, Belgium; University of Melbourne, Melbourne, Australia

**Keywords:** Cas12a/Cpf1, pre-crRNA folding, genome engineering, *Clostridium*, near-infrared fluorescence

## Abstract

*Clostridium* species have gained attention in industrial and medical applications, and the development of genetic tools has enabled the advancement of the CRISPR-Cas systems for these bacteria. Compared to the primarily used Cas9 from *Streptococcus pyogenes*, the utilization of Cas12a (previously known as Cpf1) proteins remains incomplete in clostridia, although they exhibit potential advantages, including T-rich recognition for *Clostridium* genomes and lower toxicity. In this study, we expanded the CRISPR-Cas tools in clostridia by establishing a CRISPR-Cas12a system with two different *cas12a* genes (*Ascas12a* from *Acidaminococcus* and *Fncas12a* from *Francisella novicida*). The optimized tetracycline-inducible systems were initially determined by the glucuronidase reporter and were used to drive expression of the *cas12a* genes and crRNAs. Our results demonstrate that the CRISPR-FnCas12a system offers flexible target selection in clostridia, and a specific folding pattern of the precursor crRNA is important to enable high mutation generation efficiency. By using *sacB* (encoding levansucrase) as a negative marker for plasmid curing and determining the optimal size of the donor DNA template for gene integration in the CRISPR-FnCas12a system, we achieved highly efficient and rapid genome modification, exemplified by the successful engineering of clostridia (*Clostridium butyricum* and *Clostridium sporogenes*) to produce near-infrared fluorescence from biliverdin and hemin.

**IMPORTANCE:**

Continued efforts in developing the CRISPR-Cas systems will further enhance our understanding and utilization of *Clostridium* species. This study demonstrates the development and application of a genome-engineering tool in two *Clostridium* strains, *Clostridium butyricum* and *Clostridium sporogenes*, which have promising potential as probiotics and oncolytic agents. Particular attention was given to the folding of precursor crRNA and the role of this process in off-target DNA cleavage by Cas12a. The results provide the guidelines necessary for efficient genome engineering using this system in clostridia. Our findings not only expand our fundamental understanding of genome-engineering tools in clostridia but also improve this technology to allow use of its full potential in a plethora of biotechnological applications.

## INTRODUCTION

*Clostridium* comprises anaerobic endospore-forming bacteria, some of which are industrially important (1), while others hold potential as medical vehicles (e.g., *Clostridium novyi-NT*, *Clostridium sporogenes*, and *Clostridium butyricum*), as demonstrated previously (2 – 4). To exploit their potential in biotechnological applications, the genetic tools of clostridia have been continually developed and advanced over the years (5). One such tool is the clustered regularly interspaced short palindromic repeats (CRISPR)-assisted (Cas) genome-editing system, which has been extensively studied in many organisms using various Cas proteins (6).

The Cas9 protein from *Streptococcus pyogenes* has been widely utilized for genome engineering in clostridia (7). It recognizes the DNA adjacent to the 5′-NGG-3′ protospacer adjacent motif (PAM) and results in a double-stranded break (DSB) with blunt ends (8). The low efficiency of non-homologous end joining repair in many bacteria and the lethality of unrepaired DNA necessitate the use of homology-directed repair (HDR) (9). Thus, in bacterial CRISPR systems, DNA cleavage selects for the recombination events (mediated by the donor DNA template) that repair the DSB and remove the guide RNA (gRNA) target sequence, akin to selection for resistant colonies with an antibiotic. Various methods have been employed in clostridia to overcome the toxicity of Cas9 to the host, including control of Cas9 expression using inducible promoters or a theophylline-dependent riboswitch (10), as well as selection of alternative versions of Cas9, such as the truncated Cas9 nickase (Cas9n) (7). The large size of the *cas9* gene has led some researchers to use a dual-plasmid system, which has been exemplified in solventogenic *Clostridium* species. One benefit of this approach is the improved cargo capacity of the system, which enables the insertion of larger sequences into the genome (11, 12). While genome engineering in clostridia using Cas9 has been applied extensively across multiple species, Cas12a (previously known as Cpf1) is an alternative CRISPR-associated protein that has shown potential advantages over Cas9, including the recognition of T-rich PAMs for clostridia (13) and lower toxicity (14). To date, only in a few solventogenic *Clostridium* species has the Cas12a protein been introduced for genome engineering (13, 15 – 17).

In addition to heterologous Cas proteins, which must be added to the cells as a coding sequence on a plasmid, recent publications have demonstrated the use of endogenous CRISPR-Cas systems for host genome engineering in a few *Clostridium* species. This approach relies on native expression of an endogenous Cas protein from the host chromosome, removing the need to provide the gene on a plasmid. Higher transformation efficiency is reported using this approach (18 – 22). Importantly, prior to applying the endogenous CRISPR-Cas systems, the types of CRISPR array and the requirements of PAM recognition sites in different genotypes of clostridia can vary and need to be characterized. Overall, the development of tools for genetic manipulation in clostridia has advanced significantly in the last 20 years. Further refinement of existing tools, as well as the adoption of new techniques, can support faster development of and greater control over strains for biotechnological applications (23, 24). In addition, for efficient genome engineering using CRISPR-Cas systems in bacteria, a set of guidelines for target selection and a better understanding of gRNA quality are necessary (25).

In this study, we hypothesized that the CRISPR-Cas12a system, using two tetracycline-inducible promoters for the expression of Cas12a enzymes (AsCas12a from *Acidaminococcus* and FnCas12a from *Francisella novicida*) and crRNAs, would achieve efficient genome engineering in the probiotic *C. butyricum* (26) and the oncolytic *C. sporogenes* (27). Our aim was to demonstrate the feasibility of this approach by modifying the genomes of these two *Clostridium* medical vehicles to produce near-infrared (NIR) fluorescence. Additionally, our study aimed to compare the abilities of the CRISPR-AsCas12a and CRISPR-FnCas12a systems by assessing their efficiency in generating gene (*pyrE* and *spo0A*) knockout mutations in *C. butyricum* and explore guidance for target selection in the CRISPR-Cas12a system.

## RESULTS

### Tetracycline-inducible systems regulate gene expression in *Escherichia coli* and *C. butyricum*

**Fig 1:**
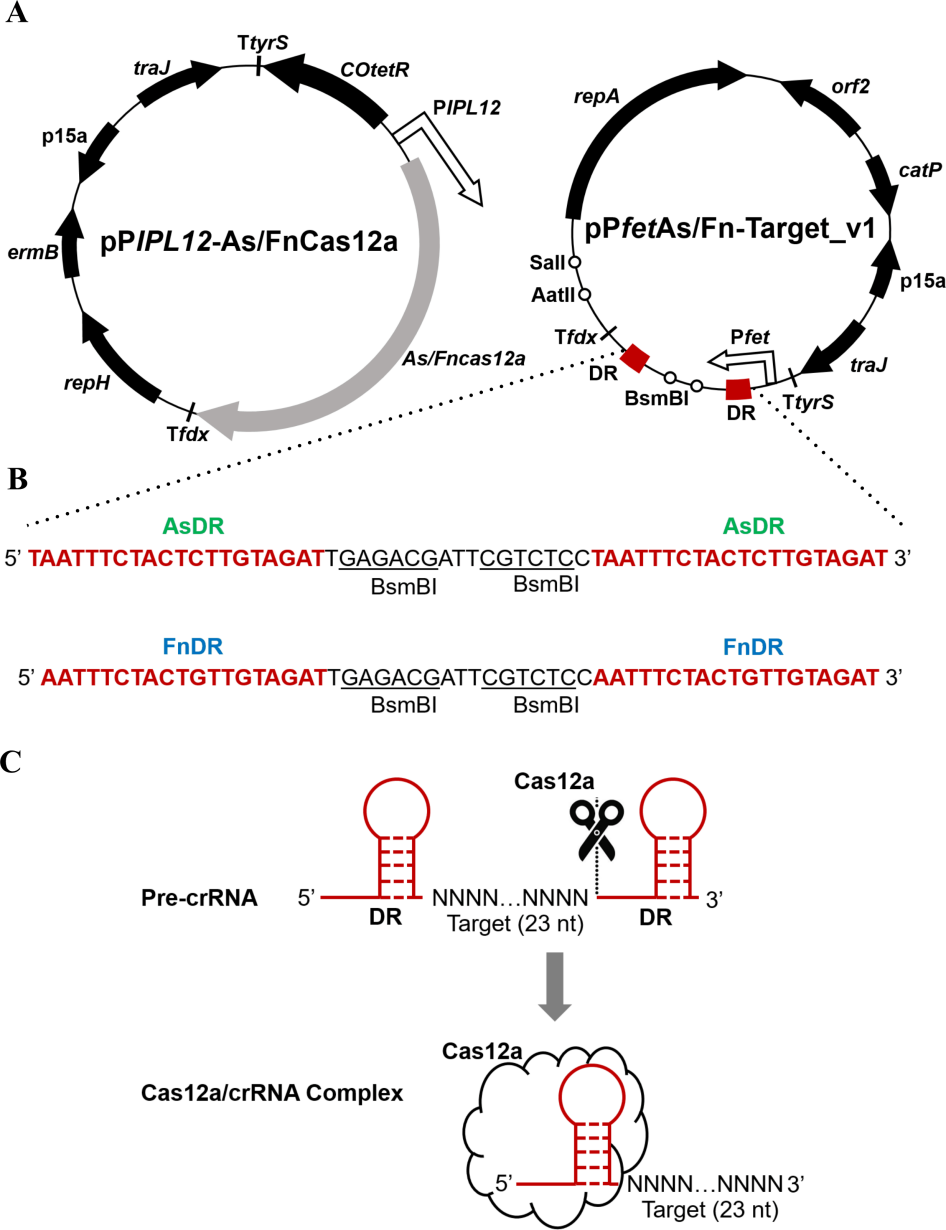
CRISPR-Cas12a systems using *Ascas12a* and *Fncas12a* genes. (**A**) Schematic representation of the vectors. (**B**) The sequence of the synthetic CRISPR array present on pP*fet*As/Fn-Target_v1. (**C**) Processing of the CRISPR array by the Cas12a enzymes. *ermB*, erythromycin resistance gene; *catP*, thiamphenicol or chloramphenicol resistance gene; p15a, Gram-negative replicon; *repH*, *orf2*, and *repA*, Gram-positive replicons; *traJ*, conjugal transfer gene; T*tyrS* and T*fdx*, terminators; DR, direct repeat sequences of the As/FnCas12a (AsDR/FnDR).

To regulate gene expression in the CRISPR system, we constructed and validated tetracycline-inducible systems in both *E. coli* and *C. butyricum* using the glucuronidase (GusA) reporter. A previous tetracycline-inducible GusA expression system (RPF185) was used as the control (28), and two optimized tetracycline-inducible promoters, P*IPL12* (29) and P*fet* (30), were evaluated. In order to align with the AT-rich genome context of clostridia, the newly codon-optimized Tet repressor (COtetR) was expressed under the control of the miniP*4*_tU promoter, resulting in the establishment of two tetracycline-inducible systems named IPL12 and fet, respectively (Fig. S1A). In the absence of the anhydrotetracycline (ATc) inducer, the GusA activity in both *E. coli gusA^−^* (Fig. S1B) and *C. butyricum* (Fig. S1C) was undetectable for all three tetracycline-inducible systems (RPF185, IPL12, and fet). However, in *C. butyricum*, the RPF185 system exhibited undetectable GusA activity even with the addition of 96-ng/mL ATc, while the GusA activity of the IPL12 and fet systems was approximately 24-fold and 31-fold higher, respectively, compared to that of the RPF185 (Fig. S1C). Based on these data, the tetracycline-inducible systems IPL12 and fet were chosen to drive expression of the *cas12a* gene and the precursor CRISPR RNA (pre-crRNA), respectively, resulting in the construction of the CRISPR-Cas12a system (CRISPR-AsCas12a and CRISPR-FnCas12a) contained in vectors pP*IPL12*-As/FnCas12a and pP*fet*As/Fn-Target_v1 (Fig. 1A). Here, the pP*fet*As/Fn-Target_v1 vector was designed to contain a CRISPR array with two direct repeat (DR) sequences and two BsmBI restriction sites, between which a 23-nucleotide target sequence can be ligated (Fig. 1B). Because the Cas12a proteins are known to process the CRISPR array by cleaving upstream of specific hairpin structures formed by the DR (31), the mature crRNA with a single 5′ repeat and the target sequence can be split from the transcript of pre-crRNA, resulting in mature Cas12a/crRNA complex (Fig. 1C).

### Optimal folding of pre-crRNA improves mutation efficiency

**Fig 2:**
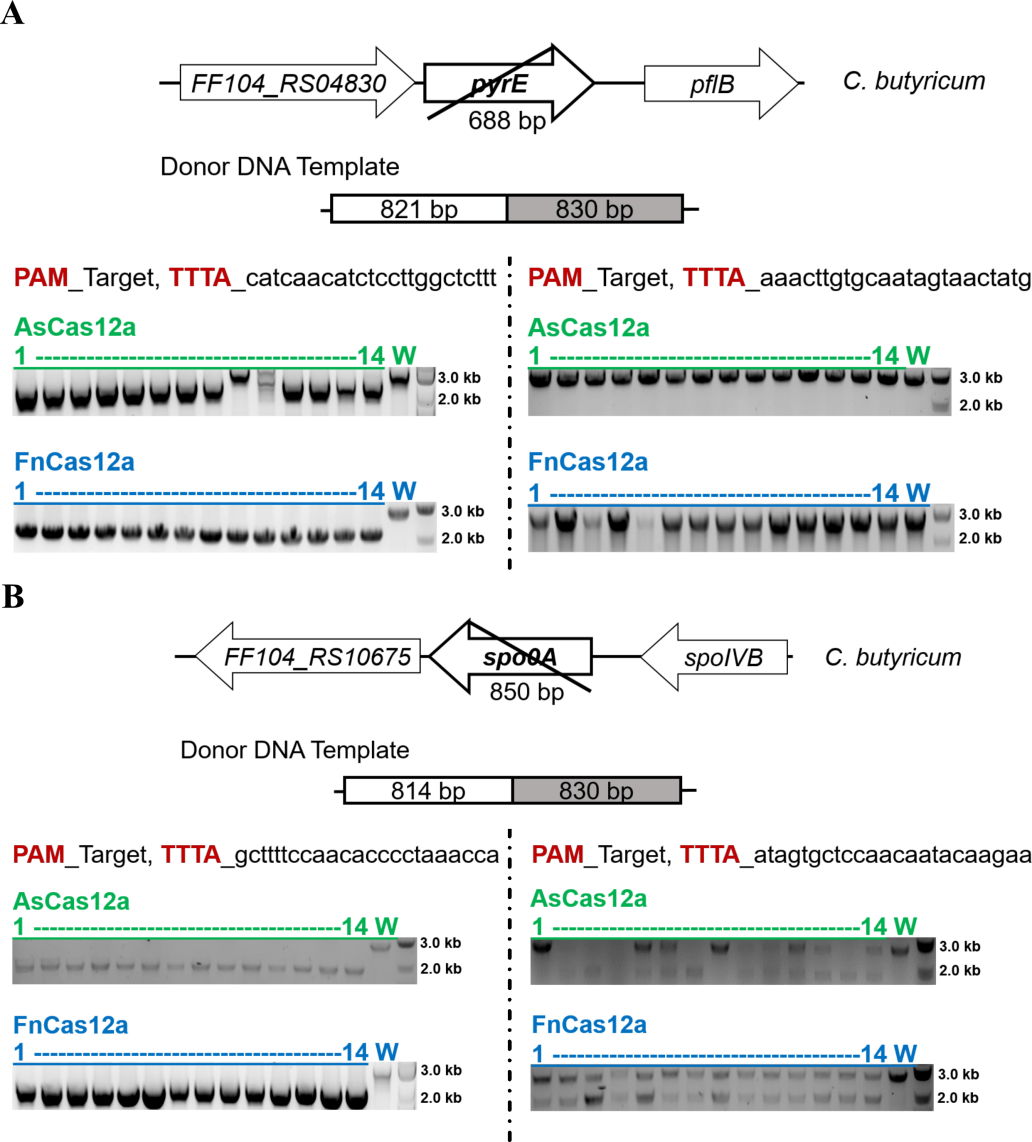
Deleting the *pyrE* and *spo0A* genes in *C. butyricum* using the CRISPR-Cas12a system. Colony PCR to check the deletions of genes *pyrE* (**A**) and *spo0A* (**B**) in *C. butyricum* by the CRISPR-AsCas12a (green) and CRISPR-FnCas12a (blue) systems. The recognized PAMs of the target sequences were highlighted in red. The PCR products of the *pyrE* mutation and the wild type were 2,172 and 2,860 bp, respectively. The PCR products of the *spo0A* mutation and the wild type were 1,901 and 2,751 bp, respectively. The Quick-Load Purple 1-kb Plus DNA (N0550L, NEB) was used as the molecular weight standard. 1–14, PCR products from single colonies; W, PCR products from the wild-type genome.

To evaluate the abilities of the CRISPR-Cas12a system, we initially deleted the *pyrE* gene (encoding orotate phosphoribosyltransferase) and the *spo0A* gene (encoding the master regulator for sporulation) in *C. butyricum* using two different targets that recognize the PAM sequence “TTTA” and using homology arms of approximately 800 bp. Using one target, both AsCas12a and FnCas12a exhibited high mutation efficiency in the deletion of the *pyrE* (Fig. 2A) or *spo0A* gene (Fig. 2B). However, another target resulted in no mutation or the presence of colonies containing both wild-type and mutant cells. This occurred in both the CRISPR-AsCas12a and the CRISPR-FnCas12a systems (Fig. 2). This result indicated that mutation efficiency is target sequence dependent for CRISPR-Cas12a systems in *C. butyricum*.

**Fig 3:**
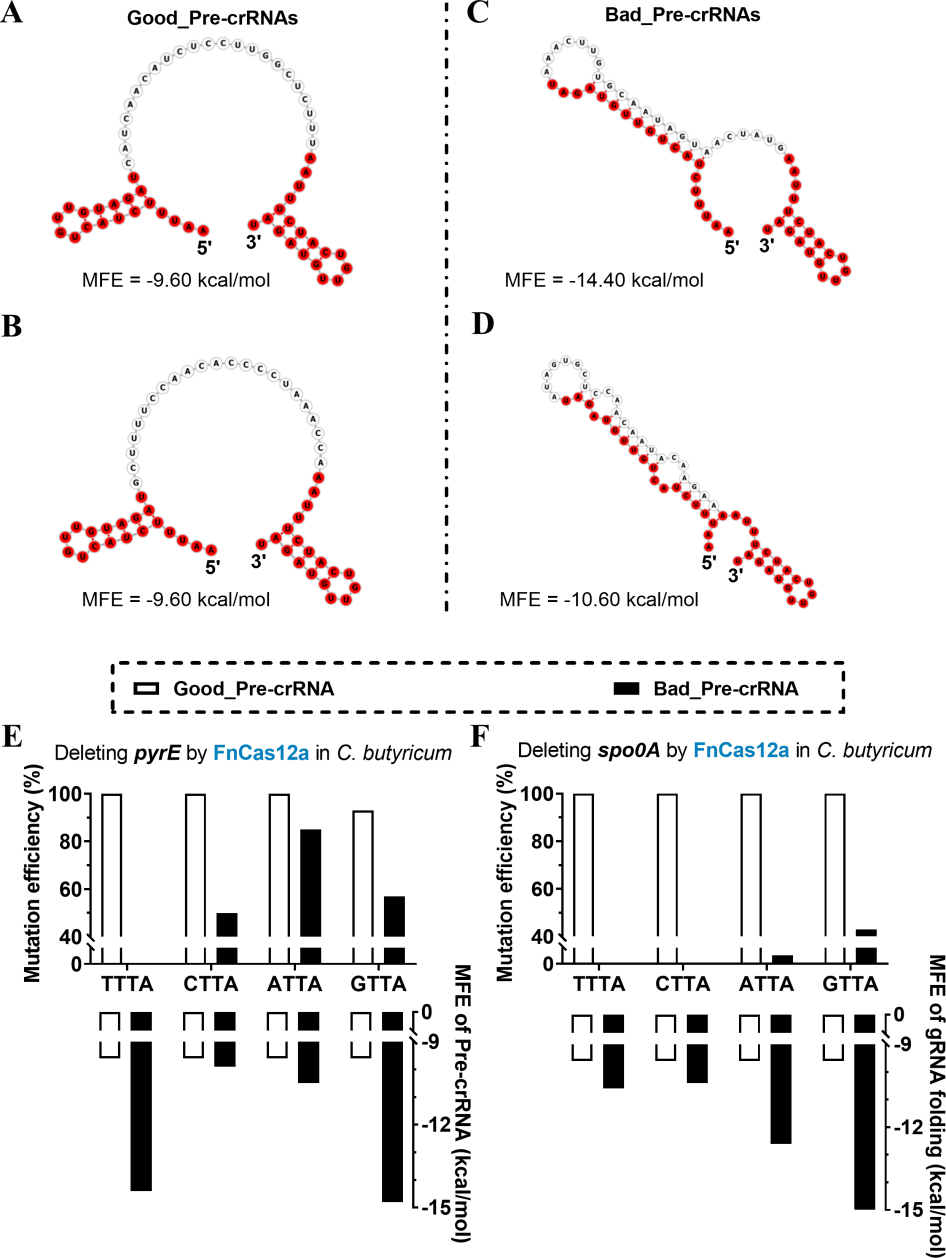
Optimal folding of pre-crRNA improves mutation efficiency. In the CRISPR-FnCas12a system, the prediction of the folding of pre-crRNAs with target sequences of (**A**) 5′-catcaacatctccttggctcttt-3′, (**B**) 5′-gcttttccaacacccctaaacca-3′, (**C**) 5′-aaacttgtgcaatagtaactatg-3′, and (**D**) 5′-atagtgctccaacaatacaagaa-3′ by the RNAfold Web Server. The mutation efficiency of deleting *pyrE* (**E**) and *spo0A* (**F**) genes in *C. butyricum* with Good_Pre-crRNAs and Bad_PrecrRNAs by the CRISPR-FnCas12a system.

In order to assess the impact of the target sequence on mutation efficiency, we utilized the RNAfold Web Server (32) to predict the folding of each pre-crRNA in the four aforementioned targets. For the CRISPR-FnCas12a system, we observed the same pre-crRNA folding (complete hairpin structures at each DR) for two target sequences that had high mutation efficiency (Fig. 3A and B). However, another two targets led to the irregular folding of pre-crRNAs, containing different base pairings between the 5′ DR and the target sequence (Fig. 3C and D), which might impair array processing and target recognition by the Cas12a proteins (33). Similar folding patterns of pre-crRNAs were observed in the CRISPR-AsCas12a system (Fig. S2). Additionally, in these same folding of pre-crRNAs, we observed the same value of −9.60 kcal/mol in minimum free energy (MFE), which represents the most thermodynamically stable structure of an RNA molecule under certain conditions (32). We hypothesized that with the MFE value of −9.60 kcal/mol, the possibly optimal folding structure of the pre-crRNA contributed to the high mutant efficiency in the CRISPR-Cas12a system. Thus, each pre-crRNA formed by different targets and predicted with −9.60 kcal/mol in MFE was defined as the Good_Pre-crRNA, while one without such an MFE was defined as the Bad_Pre-crRNA.

Using the CRISPR-FnCas12a system with various targets recognizing different PAMs, all Good_Pre-crRNAs displayed above 90% mutation efficiency in both the *pyrE* and *spo0A* gene deletions in *C. butyricum*. However, the Bad_Pre-crRNAs resulted in variable mutation efficiency in the deletions of *pyrE* and *spo0A* in *C. butyricum* (ranging from 0% to 80%, Fig. 3E and F). No clear correlation was observed between the MFE of the Bad_Pre-crRNAs and the mutation efficiency, suggesting that other factors in the Bad_Pre-crRNA may also affect mutation efficiency. Hence, the folding of the pre-crRNA can affect the mutation efficiency, and the prediction of Good_Pre-crRNA could be beneficial for the use of the CRISPR-Cas12a system in *C. butyricum*.

To validate this finding across species, the CRISPR-FnCas12a system was utilized to delete the *uidA* gene (encoding beta-glucuronidase) in *E. coli* MG1655 RARE (34), using a Good_Pre-crRNA and a Bad_Pre-crRNA recognizing a PAM of ATTA. Similar to the results in *C. butyricum*, the Good_Pre-crRNA displayed 100% mutation efficiency, while the Bad_Pre-crRNA resulted in most colonies (six out of 14 colonies) containing a mixture of wild-type and mutation cells (Fig. S3). This finding supports the notion that the folding of Good_Pre-crRNA generates high mutant efficiency using the CRISPR-FnCas12a system in a species-independent manner.

### CRISPR-FnCas12a is an efficient and flexible gene deletion system in clostridia

**Fig 4:**
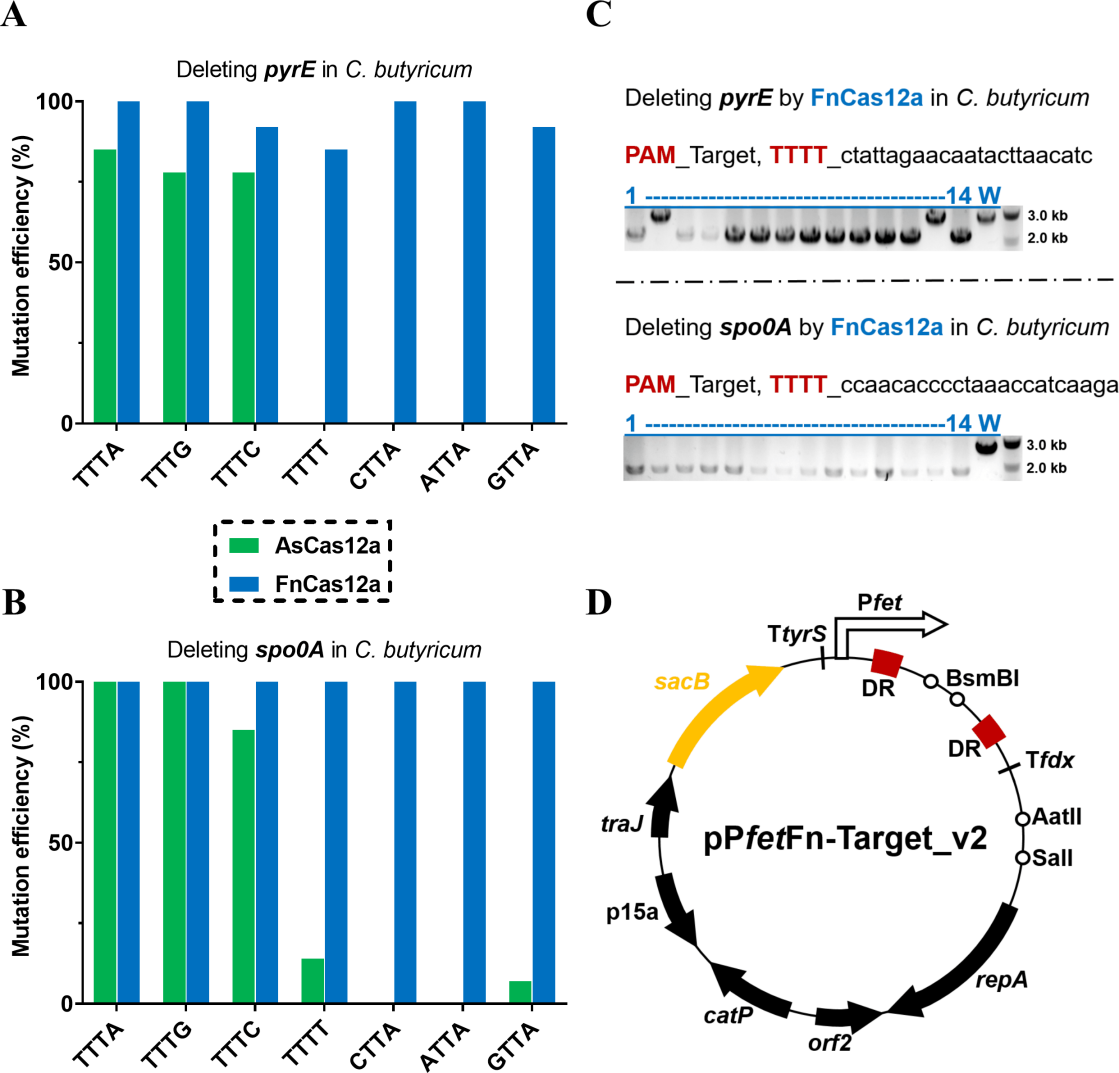
Efficient and flexible gene deletion in clostridia by the CRISPR-FnCas12a system and the inclusion of the SacB-negative marker for efficient plasmid curing. Using various Good_Pre-crRNAs recognizing different PAMs, the mutation efficiency of deleting *pyrE* (**A**) and *spo0A* (**B**) genes in *C. butyricum* by the CRISPR-AsCas12a (green) and CRISPR-FnCas12a (blue) systems was determined. (**C**) Using targets recognizing TTTT PAM and colony PCR to check deletions of *pyrE* and *spo0A* genes in *C. butyricum* by the CRISPR-FnCas12a system. (**D**) The schema of the general vector pP*fet*Fn-Target_v2. The inclusion of the SacB negative marker was highlighted in yellow.

Upon the findings about the Good_Pre-crRNA, to compare the PAM recognition between the CRISPR-AsCas12a and CRISPR-FnCas12a systems, various targets forming the Good_Pre-crRNA in the two CRISPR-Cas12a systems were used to delete the *pyrE* and *spo0A* genes in *C. butyricum*. Using the Good_Pre-crRNA, the CRISPR-AsCas12a system showed high mutation efficiency with PAMs of TTTA, TTTG, and TTTC but low efficiency with TTTT, CTTA, ATTA, and GTTA (Fig. 4A and B), which is in accordance with the known preference of AsCas12a for PAMs of TTTV (V = A/G/C) (35). It has been recently observed that the cleavage efficiency of FnCas12a decreases significantly with a TTTT PAM in several species (36, 37). However, our CRISPR-FnCas12a system showed high mutation efficiency when deleting either the *pyrE* or *spo0A* genes not only when using PAMs of TTTA, TTTG, TTTC, CTTA, ATTA, or GTTA (Fig. 4A and B) but also when using the TTTT PAM (Fig. 4C). Thus, we concluded that in clostridia, TTN PAM recognition by the CRISPR-FnCas12a system was efficient. The CRISPR-FnCas12a system was chosen for subsequent experiments as it enabled maximum flexibility in the selection of targets that formed the Good_Pre-crRNA in the AT-rich genomes of *Clostridium* species.

### The *sacB* gene can be used as a negative marker for plasmid curing

To characterize phenotypes following confirmation of the mutation through colony PCR, the CRISPR plasmids involved in the mutation should be cured. We initially observed that the variations of plasmid pP*fet*Fn-Target_v1 were not efficiently curable in *C. butyricum* (data not shown). To enhance plasmid-curing efficiency in the CRISPR-FnCas12a system, the *sacB* gene (encoding levansucrase) was cloned into pP*fet*Fn-Target_v1 as a negative marker (38), resulting in plasmid pP*fet*Fn-Target_v2 (Fig. 4D). The addition of 100-g/L sucrose to the cultures containing both the pP*IPL12*-FnCas12a vector and the derivative vector based on pP*fet*Fn-Target_v2 after overnight incubation did not result in any colonies on agar plates containing the antibiotics thiamphenicol (Tm) or chloramphenicol (Cm), indicating successful curing of the derivative of pP*fet*Fn-Target_v2. On the other hand, colonies containing the pP*IPL12*-FnCas12a vector could be selected on an agar plate supplemented with the antibiotic erythromycin (Erm) and used for further editing. Similar results were observed in *C. butyricum*, *C. sporogenes*, and *E. coli* (Fig. S4). Thus, the inclusion of the *sacB*-negative marker was found to be effective in promoting plasmid curing in the CRISPR-FnCas12a system.

### CRISPR-FnCas12a-mediated gene deletions result in corresponding phenotype changes

In order to evaluate whether CRISPR-mediated gene deletions resulted in the expected outcome, we characterized the corresponding phenotypes of the mutations. For the characterization of the *pyrE* gene deletion, we evaluated cell growth in the presence of 5-fluoroorotic acid (FOA). This approach is based on the observation that FOA is toxic to clostridia harboring a functional *pyrE* pathway (5). As expected, *C. butyricum* harboring the deletion of *pyrE* (CB_Δ*pyrE*) was able to grow on agar plates supplemented with 1-mg/mL 5-FOA, whereas *C. butyricum* wild type (CB_WT) was unable to grow due to the presence of the *pyrE* pathway (Fig. S5A). For the characterization of the *spo0A* gene deletion, we performed a sporulation assay. Using this assay, we demonstrated that *C. butyricum* harboring the *spo0A* deletion (CB_Δ*spo0A*) was unable to form spores, whereas the CB_WT strain was able to generate heat-resistant colonies of about 5 × 10^5^ CFU/mL (Fig. S5B). Additionally, in the GusA assay, *E. coli* MG1655 RARE harboring the deletion of *uidA* (RARE_Δ*uidA*) was unable to exhibit detectable GusA activity, while the wild-type strain RARE_WT could (Fig. S5C). These results demonstrate that gene deletions using the CRISPR-FnCas12a system lead to corresponding phenotype changes.

### Gene integration efficiency is affected by the size of homology arms in donor DNA templates

**Fig 5:**
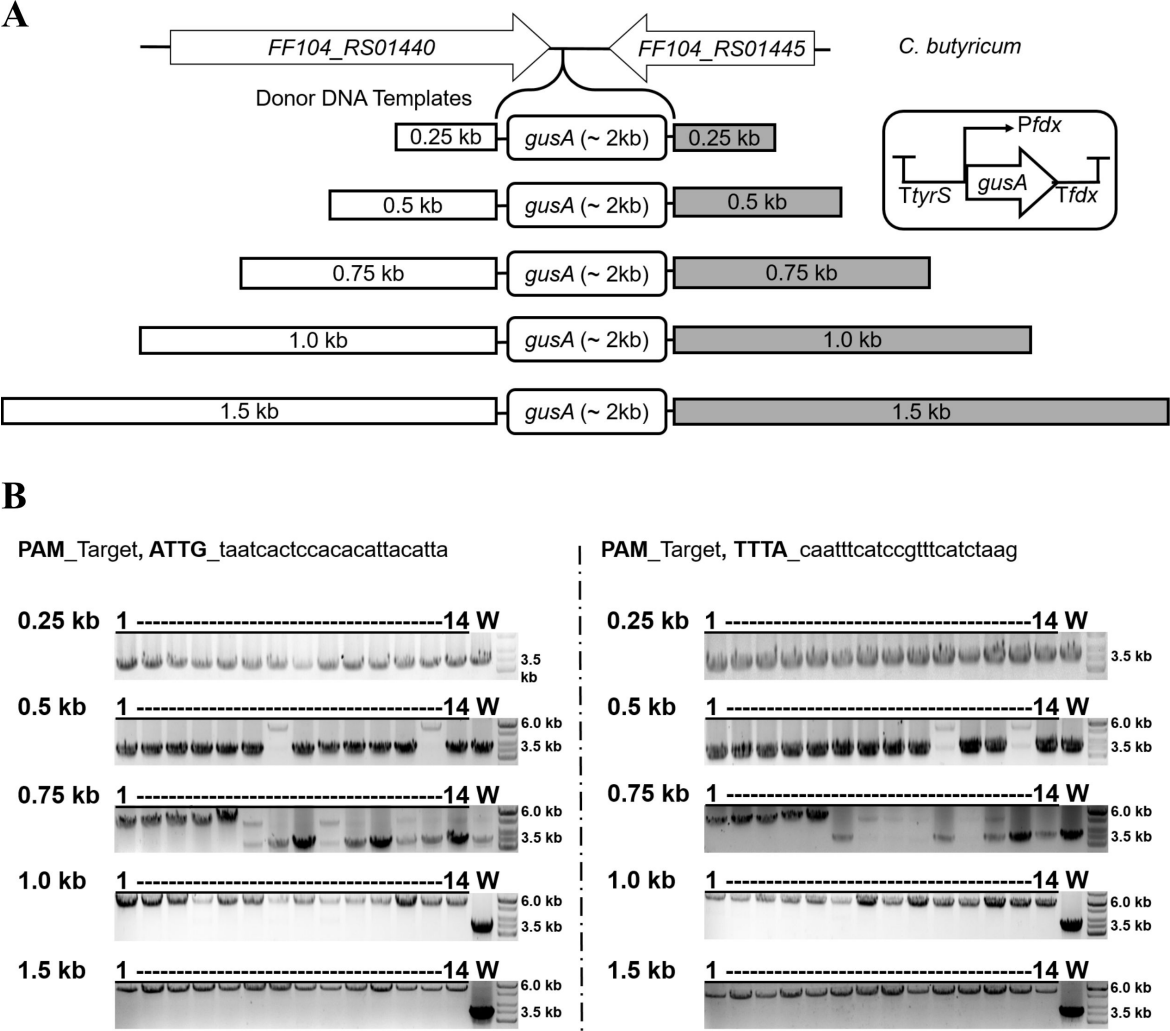
Donor DNA templates affect gene integration efficiency. (**A**) The schema of donor DNA templates with different sizes of homology arms to integrate the GusA expression cassette (about 2-kb size) into *C. butyricum*. (**B**) In the CRISPR-FnCas12a system, use colony PCR to check the integration of the GusA expression cassette in *C. butyricum* using two different targets. The sizes of the PCR products of the mutation harboring the integration of the GusA expression cassette and the wild type were 5,426 and 3,536 bp, respectively. The GeneRuler 1-kb DNA ladder (SM0311, Thermo Scientific) was used as the molecular weight standard. 1–14, PCR products from single colonies; W, PCR products from the wild-type genome.

The efficiency of recombination of the donor DNA template is a crucial factor in HDR-mediated CRISPR systems (39), and the appropriate size of homology arms may vary depending on the species or systems. In the experiments of gene deletion carried out in *E. coli* and *C. butyricum* as described above, a 0.8-kb homology arm was selected. Using the CRISPR-FnCas12a system, to further examine the effect of homology arm size on recombination efficiency in *C. butyricum*, we truncated the size of the homology arm in the donor DNA template from 1.5 to 0.25 kb (Fig. 5A). Using a 0.25-kb size of homology arm to integrate a 2-kb size of *gusA* expression cassette into *C. butyricum* did not result in the desired mutation. The mutation was observed when the homology arm size was increased from 0.5 to 1.5 kb. The mutation efficiency reached 100% with 1.0- and 1.5-kb homology arms. Similar results were obtained using different targets (Fig. 5B). These data indicate that the efficiency of gene integration using the CRISPR-FnCas12a system is dependent on the homology arm size in the donor DNA template and that a size of at least 0.5 kb is necessary for the recombination to acquire the desired mutation in *C. butyricum*. Of note, using the GusA assay, we characterized the mutation phenotype of *C. butyricum* harboring the integrated *gusA* expression cassette (CB_IN*gusA*), which showed an increase in GusA activity compared to CB_WT (Fig. S5C).

### *C. butyricum* and *C. sporogenes* can be engineered to produce NIR fluorescence

**Fig 6:**
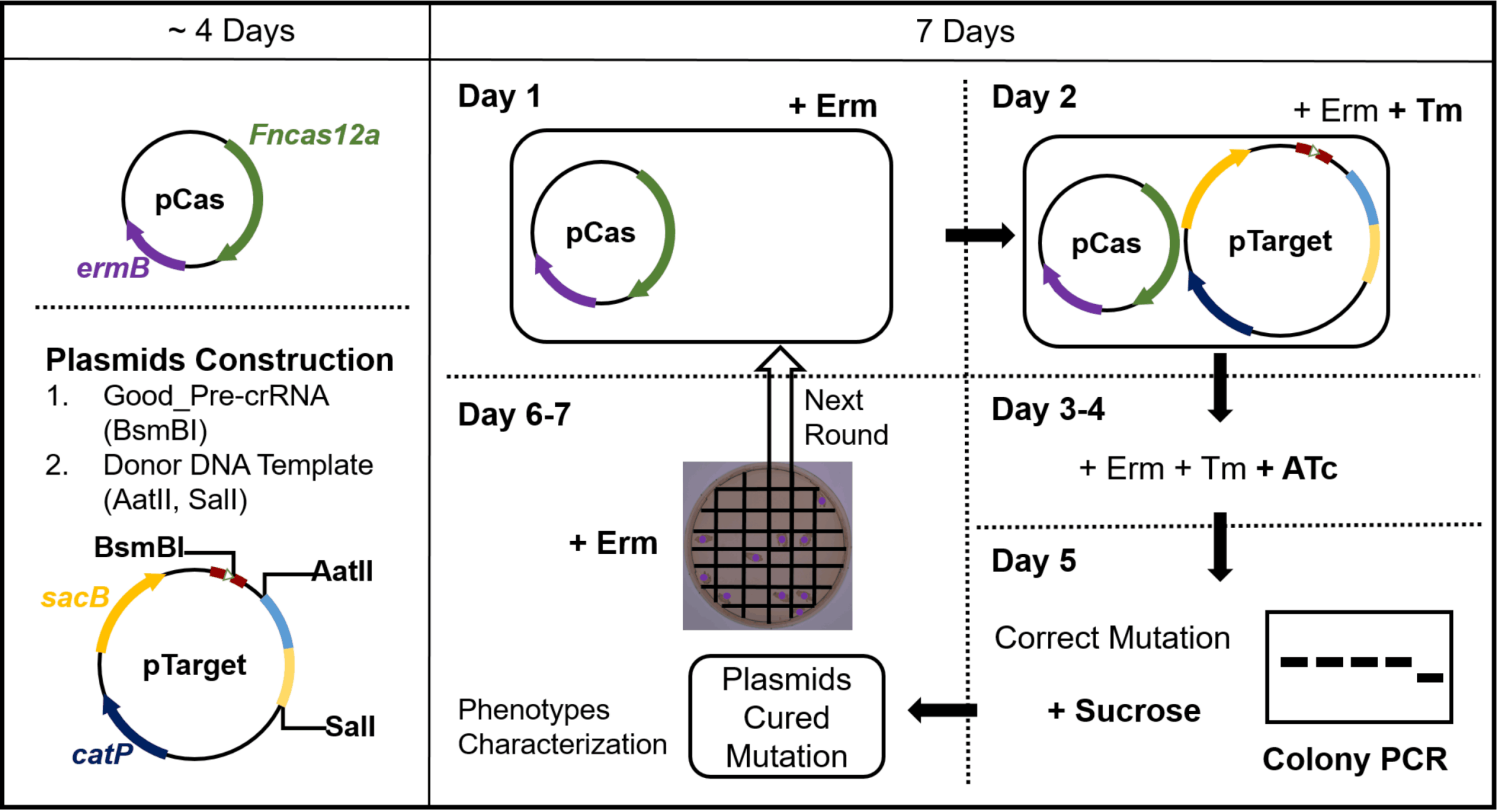
Procedure for genome engineering in clostridia by the CRISPR-FnCas12a system. The targets and donor DNA templates are ligated into pTarget (the general vector pPfetFn-Target_v2) and confirmed by Sanger sequencing (4 days). The engineered clostridia are generated as follows: the confirmed vector pTarget is transformed into the clostridia containing pCas (the pP*IPL12*-FnCas12a vector) (Days 1 and 2); the trans-conjugants are re-streaked on new agar plates containing the ATc inducer (Days 3 and 4); the correct mutations are confirmed by colony PCR and are cultures overnight with the addition of sucrose for plasmids curing (Day 5); an overnight culture of the correct mutations is serially diluted and plated (Day 6); the presence of each plasmid in mutants is determined by their ability to grow on antibiotic selection plates (Day 7). Clones that can grow on Erm plates are used for subsequent rounds of genetic engineering (Fig. S4). The desired changes to the genome are confirmed by Sanger sequencing; furthermore, the phenotype can be functionally characterized. Erm, 15-µg/mL erythromycin; Tm, 15-µg/mL thiamphenicol; ATc, 96-ng/mL anhydrotetracycline; Sucrose, 100-g/L sucrose.

As depicted in Fig. 6, the construction of plasmids following the guidelines for target selection and donor DNA template design can be accomplished within about 4 days. Subsequently, genome-engineered clostridia can be generated using the CRISPR-FnCas12a system within about 1 week, and then the phenotype of the plasmid-cured mutations can be characterized.

**Fig 7:**
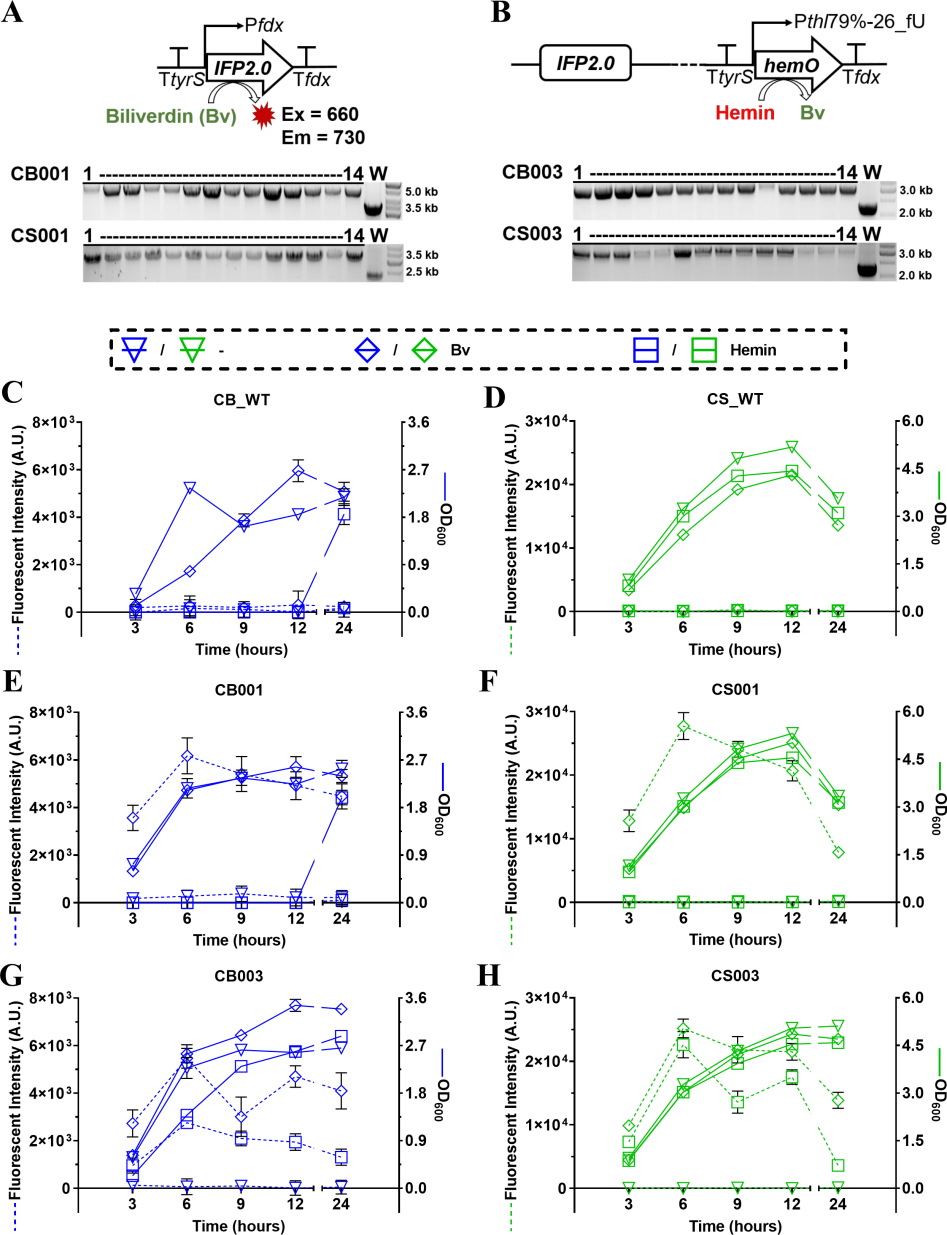
Genome engineering in *C. butyricum* and *C. sporogenes* to produce NIR fluorescence from Bv and hemin. (**A**) Colony PCR to check the integration of the IFP2.0 expression cassette in wild-type strains of *C. butyricum* and *C. sporogenes*, obtaining CB001 (size of mutation/wild type, 4,580/3,536 bp) and CS001 (size of mutation/wild type, 3,506/2,475 bp) strains. (**B**) Colony PCR to check the integration of the HemO expression cassette with promoter P*thl*79%-26_fU in CB001 and CS001 strains, obtaining CB003 (size of mutation/wild type, 3,133/2,338 bp) and CS003 (size of mutation/wild type, 3,119/2,343 bp) strains. With or without the addition of Bv or hemin, the growth curve and the intensity of NIR fluorescence (A.U. in 660/730 nm of Ex/Em) in wild type CB_WT (**C**) and CS_WT (**D**) strains and the CB001 (**E**), CS001 (**F**), CB003 (**G**), and CS003 (**H**) strains. Bv, 10 µM in all strains; Hemin, 7.5 µM in CB_WT, CB001, and CB003 strains; and 2.5 µM in CS_WT, CS001, and CS003 strains. The data represent the mean ± SD of three biological replicates.

To demonstrate the versatility and efficiency of the tetracycline-inducible CRISPR-FnCas12a system across different *Clostridium* species, we set out to engineer *C. butyricum* and *C. sporogenes* to produce NIR fluorescence, which might offer a deep penetration in the anaerobic tracking of clostridia (40). The *IFP2.0* gene, derived from *Rhodopseudomonas palustris*, was used as it codes for a NIR fluorescent protein that requires biliverdin (Bv) as a chromophore cofactor (41). The *IFP2.0* gene expression cassette was codon optimized and efficiently integrated into the genomes of *C. butyricum* (CB001 strain) and *C. sporogenes* (CS001 strain), as shown in Fig. 7A.

**Fig 8:**
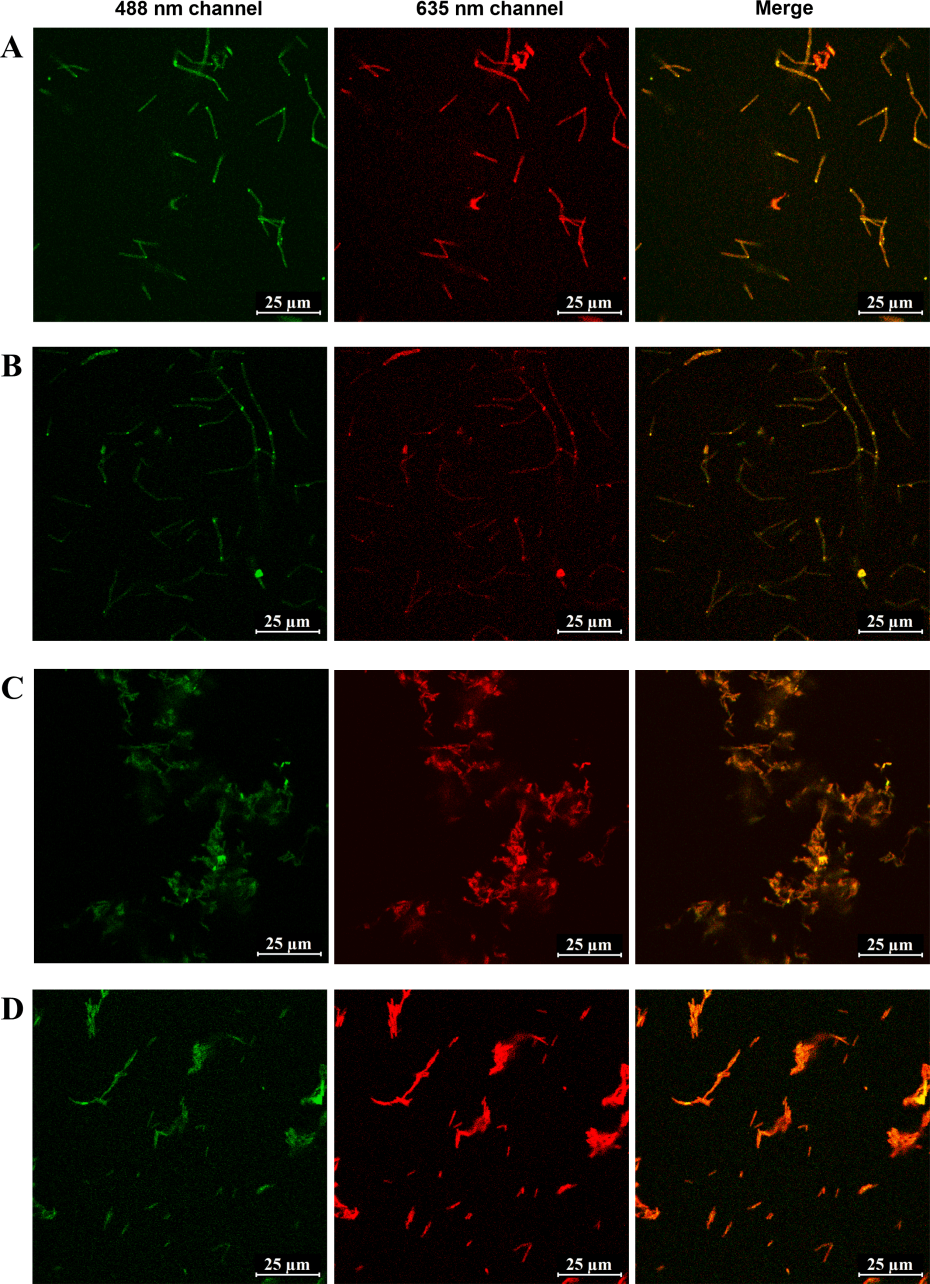
Confocal imaging in 488 635 nm and merged channels of the CB003 and CS003 strains with the addition of Bv or hemin. (**A**) The CB003 with an addition of 10-µM Bv; (**B**) the CB003 with an addition of 7.5-µM hemin; (**C**) the CS003 with an addition of 10-µM Bv; and (**D**) the CB003 with an addition of 2.5-µM hemin. Scale bar, 25 µm.

The green bile pigment, Bv, is catabolized from hemin by the *hemO* gene (encoding hemin oxygenase). The *hemO* gene from *C. novyi*, driven by a strong promoter P*thl*79%−26_fU, was codon optimized and efficiently integrated into the genomes of the CB001 and CS001 strains, resulting in the CB003 and CS003 strains, respectively (Fig. 7B). The wild-type CB_WT and CS_WT strains did not exhibit detectable NIR fluorescence, with or without the addition of either Bv or hemin (Fig. 7C and D). The CB001 and CS001 strains showed NIR fluorescence only with the addition of 10-µM Bv, and the highest fluorescence intensity was observed at around the 6-hour time point (Fig. 7E and F). Due to the toxicity of high concentrations of hemin in bacteria (42), we determined the appropriate concentration of hemin in the CB003 and CS003 strains (Fig. S6). With the addition of 7.5-µM hemin, the CB003 strain displayed NIR fluorescence along with cell growth (Fig. 7G), while the CB_WT and CB001 could not exhibit obvious growth until the 24-hour time point without NIR fluorescence (Fig. 7C and E). These data suggest that the integration of hemin oxygenase could improve the tolerance of hemin toxicity in *C. butyricum*. Furthermore, with the addition of 2.5-µM hemin, the CS003 strain displayed NIR fluorescence along with normal cell growth (Fig. 7H). When supplied with either hemin or Bv, at the 6-hour time point, confocal imaging revealed NIR fluorescent cells in the CB003 and CS003 strains (Fig. 8), while only green auto-fluorescent cells were observed in the wild-type strains (Fig. S7). These data demonstrate that the genomes of *C. butyricum* and *C. sporogenes* could be modified by the tetracycline-inducible CRISPR-FnCas12a to produce NIR fluorescence from hemin and Bv.

## DISCUSSION

*Clostridium* offers a broad range of potential applications in biotechnology, whether for medical or industrial applications. However, genetic manipulation in clostridia is still challenging due to species-specific nuances (43), and the CRISPR-Cas tools in clostridia are not as refined as in model organisms (7). In this study, we achieved successful implementation of the CRISPR-Cas12a system in two *Clostridium sensu stricto* species, *C. butyricum*, and *C. sporogenes*, utilizing tetracycline-inducible systems.

Currently, many *Clostridium* studies have designed multiple single-guide RNAs (sgRNAs) and gRNAs in the CRISPR-Cas systems to acquire mutations efficiently, which is limited for the rapid genome engineering in *Clostridium* research (7). Unlike the sgRNA selection in CRISPR-Cas9 systems by various computational tools (25), the identification of gRNAs with high mutation efficiency remains a challenge for CRISPR-Cas12a systems in diverse species (31, 44, 45). Initially, we used the web tool CRISPOR (46) to select target sites for pre-crRNA, with TTTV as the PAM for both AsCas12a and FnCas12a. Despite this effort, some targets still exhibited low efficiency in mutation generation. Inspired by the pre-crRNA processing of Cas12a, we determined that predicting the specific folding of pre-crRNA by utilizing the RNAfold Web Server (32) could enhance the mutation efficiency in *C. butyricum*. By deleting the *uidA* gene in the model microbe *E. coli*, we suggest that selecting targets based on pre-crRNA folding predictions may improve the efficiency of CRISPR-Cas12a systems across various species. There is a recent observation in *Clostridium beijerinckii* that some target sequences in the CRISPR array result in the low efficiency of their CRISPR-FnCas12a system (17).

In previous studies that focused on the impact of PAM selection on Cas12a-mediated genome engineering, the design of the CRISPR array was different and pre-crRNA folding was not analyzed in detail. This could explain inconsistent reports on the performance of FnCas12a when “TTT” is selected as the PAM sequence. The widespread availability of “TTN” as a PAM sequence in *Clostridium* genomes increases the number of loci that can be targeted but may also raise concerns over off-target effects (47). Predicting the folding of pre-crRNA should be a necessary aspect to be considered in *Clostridium* CRISPR-Cas12a systems, to carefully select targets and reduce potential off-target effects. Furthermore, high-GC targets have been found to impair the activity of the Cas12a array (31). Given the low GC content in clostridia genomes, we used targets with an average GC content of below 50% (Table 1), which may not be a critical aspect of exploring CRISPR-Cas12a systems in clostridia. However, we encountered difficulty determining why some targets forming the “bad” folding of pre-crRNA were still efficient in our study. We hypothesize that the frequency of “bad” folding in pre-crRNAs could differ due to the varying stability of base pairing (48), resulting in on-target effects of Bad_Pre-crRNAs. Therefore, the accurate prediction of RNA folding would be beneficial for investigating off-target effects, and we encourage RNA folding to be considered in computational tools to predict efficient gRNAs (25). Recently, several dCas12a-based CRISPR interference systems have been developed for tuning gene expression in clostridia (13, 47, 49). In our later work, we would like to investigate the impact of the folding of pre-crRNAs on the efficiency of gene repression.

**TABLE 1:** Targets used in the CRISPR-FnCas12a system

PAMs	5′–3′ sequences	GC content	MFE of pre-crRNA (kcal/mol)	Mutation efficiency (%)	Description
TTTA	catcaacatctccttggctcttt	0.43	9.60	100	Forming Good_Pre-crRNAs to delete the *pyrE* gene in *C. butyricum*
CTTA	tctccatcatttatcttacttcc	0.35	9.60	100	
ATTA	taagcccttttacatcaacatct	0.35	9.60	100	
GTTA	ctattgcacaagttttaaaacca	0.30	9.60	93	
TTTG	taacaaaatctccgaatgtcaac	0.35	9.60	100	
TTTC	atcttttattcctccgttaactt	0.30	9.60	93	
TTTT	ctattagaacaatacttaacatc	0.26	9.60	86	
TTTA	aaacttgtgcaatagtaactatg	0.30	14.40	0	Forming Bad_Pre-crRNAs to delete the *pyrE* gene in *C. butyricum*
CTTA	cttcctaacaagatccctttatc	0.39	9.90	50	
ATTA	gttgaaataagagaaaaatttgg	0.26	10.50	86	
GTTA	caacagcaggaacttcgatttat	0.39	14.80	57	
TTTA	gcttttccaacacccctaaacca	0.48	9.60	100	Forming Good_Pre-crRNAs to delete the *spo0A* gene in *C. butyricum*
CTTA	tccaacaacttcttcatatattc	0.30	9.60	100	
ATTA	caaacataattcatgaaatcggt	0.30	9.60	100	
GTTA	ttcttgatatcataatgccacat	0.30	9.60	100	
TTTG	caatccccgtaaccacaatatct	0.43	9.60	100	
TTTC	caacacccctaaaccatcaagat	0.43	9.60	100	
TTTT	ccaacacccctaaaccatcaaga	0.48	9.60	100	
TTTA	atagtgctccaacaatacaagaa	0.35	10.60	0	Forming Bad_Pre-crRNAs to delete the *spo0A* gene in *C. butyricum*
CTTA	ttctttttgtaaatacttccatg	0.26	10.40	0	
ATTA	attgcttctatttgtcctctgcc	0.43	12.60	21	
GTTA	cggggattgcaaaagatggtaga	0.48	15.00	43	
ATTA	accacaaaccgttctactttact	0.39	9.60	100	Forming Good_Pre-crRNA to delete the *uidA* gene in *E. coli* MG1655 RARE
ATTA	cgctgcgatggatcccggcatag	0.65	15.00	21	Forming Bad_Pre-crRNA to delete the *uidA* gene in *E. coli* MG1655 RARE
ATTG	taatcactccacacattacatta	0.30	9.60	100	Forming Good_Pre-crRNAs to integrate the *gusA*/*IFP2.0* gene expression cassette into *C. butyricum*
TTTA	caatttcatccgtttcatctaag	0.35	9.60	100	
TTTC	tactttcaatctcatataaaaaa	0.17	9.60	100	Forming Good_Pre-crRNAs to integrate the *IFP2.0* gene expression cassette into *C. sporogenes*
CTTG	ggaaagaaagaatctaagaagaa	0.30	9.60	100	Forming Good_Pre-crRNAs to integrate the *hemO* gene expression cassette with promoter P*thl*_fU/P*thl*79%−26_fU into *C. butyricum*
TTTA	aaagactgtctcaaaataaaaat	0.22	9.60	100	Forming Good_Pre-crRNAs to integrate the *hemO* gene expression cassette with promoter P*thl*_fU/P*thl*79%−26_fU into *C. sporogenes*
TTTG	aacttaatttttatatcaatcta	0.13	9.60	93	Forming Good_Pre-crRNAs to integrate the *hemLA* polycistronic expression cassette into *C. sporogenes*
GTTG	tctcaaagtaaaaatagatttaa	0.17	9.60	86	Forming Good_Pre-crRNAs to integrate the *hemNG* polycistronic expression cassette into *C. sporogenes*
TTTA	tattctcttattttgatacaatt	0.17	9.60	100	Forming Good_Pre-crRNAs to integrate the *hemEH* polycistronic expression cassette into *C. sporogenes*

Prior to engineering the genome of clostridia for NIR fluorescence production, we evaluated three oxygen-independent fluorescent proteins: CreiLOV (50), UnaG (51), and IFP2.0 (41), which use natural substrates as cofactors (flavin, bilirubin, and Bv, respectively). However, during confocal imaging in the 488-nm channel, we observed high levels of green auto-fluorescence in *C. butyricum*, which made it difficult to distinguish between *C. butyricum* expressing CreiLOV or UnaG with and without the addition of flavin or bilirubin. In contrast, *C. butyricum* expressing IFP2.0 exhibited low background auto-fluorescence in the 635-nm channel, making it easier to distinguish the addition of Bv (Fig. S7). The green auto-fluorescence has been observed in *Clostridioides difficile*, the mechanism of which is still unclear (52). In addition, various Bv-based red fluorescent proteins have been developed continuously (53), which would serve as alternatives to IFP2.0. After integrating IFP2.0, we initially integrated the *hemO* gene driven by the promoter P*thl*_fU into the CB001 and CS001 strains, obtaining the CB002 and CS002 strains. We observed that hemin led to growth inhibition in the CB002 and CS002 strains (Fig. S6), and, thus, we improved the expression of the *hemO* gene by substituting the promoter P*thl*_fU with a stronger promoter, P*thl*79%-26_fU (38, 54). The toxicity of hemin in bacteria is multifactorial, and our understanding of it is still incomplete (42, 55). In some *Clostridium* species, hemin oxygenase is involved in enhanced aero-tolerance by degrading hemin to Bv (56), which could improve the colonization capacity in *Clostridium*-mediated therapy (3). In order to avoid the external supply of hemin, we attempted to integrate the hemin biosynthetic pathway into the genome of *C. sporogenes* using the CRISPR-FnCas12a system (Fig. S8A). Inspired by the metabolic engineering of *E. coli* for hemin production (57, 58), we introduced three polycistronic expression cassettes into the genome of the CS003 strain with high mutation efficiency: *hemLA* (2,766 bp), *hemNG* (2,212 bp), and *hemEH* (2,320 bp) resulting in the creation of strain CS006 (Fig. S8B). Despite the addition of the required Fe^2+^ ion and the electron acceptor menaquinone of HemG (59), the final strain CS006 did not exhibit detectable NIR fluorescence along with cell growth (Fig. S8C and D). This may be due to a lack of metabolic flux into the hemin biosynthetic pathway. Overexpression of critical genes to increase the metabolites available for this pathway could provide a solution. This could be achieved by increasing the copy number of critical genes in the genome or on a high copy number plasmid, as previously reported (60). However, to date, there are no reports of *de novo* hemin biosynthesis in clostridia. The lack knowledge on the biology of non-model microbes like clostridia, especially when compared to *E. coli*, presents a major challenge for metabolic engineering and synthetic biology (24). Overall, this study demonstrates that the CRISPR-FnCas12a system can be a powerful gene integration tool in clostridia. This work introduces the concept of endogenous fluorescence in recombinant clostridia, which will enable visualization and tracking of the bacteria. The substrate for the fluorescence reaction (hemin) occurs naturally and can be obtained from dietary sources and from hemoglobin and Bv, a pigment that produced in the intestine. This is an obvious advantage for the application of genetically engineered clostridia in the medical setting, such as intestinal probiotics and intratumoral living therapeutics (3, 61, 62).

In conclusion, this study introduces a tetracycline-inducible CRISPR-Cas12a system that enables robust genome engineering in two *Clostridium* medical vehicles. We achieved gene deletions and heterologous gene integrations with a high efficiency ranging from 85% to 100% using the CRISPR-FnCas12a system (Table 1), which can be one of the most powerful CRISPR-Cas systems in clostridia. The study highlights the significance of RNA folding prediction in analyzing the off-target effects of Cas12 proteins in various species. Further studies on the NIR fluorescent clostridia provide an opportunity for anaerobic tracking in *Clostridium*-mediated living therapeutics and emphasize the importance of understanding this non-model microbe for biotechnological applications using genome-engineering tools.

## MATERIALS AND METHODS

### Strains and growth conditions

All details of the used strain are listed in Table S1. Three *E. coli* strains (10-beta, *gusA^−^*, and S17-1) were used for vector cloning, the GusA reporter assay, and bacterial conjugation, respectively. *E. coli* strain MG1655 RARE was obtained from Addgene (#61440). All *E. coli* strains containing plasmids were grown routinely in lysogeny broth (LB) or 1.5% (wt/vol) agar plates supplemented with appropriate antibiotics (12.5- to 25-µg/mL Cm; 500-µg/mL Erm). Two *Clostridium* strains (*C. sporogenes* NCIMB 10696 and *C. butyricum* DSM10702) were grown in peptone yeast thioglycolate (PYT) media with the addition of 10-g/L D-glucose (PYTG) or 1.5% (wt/vol) agar plates (38), supplemented with appropriate antibiotics (250-µg/mL D-cycloserine, 15-µg/mL Tm, and 15-µg/mL Erm) or inducer (32- or 96-ng/mL ATc) as necessary. In addition, PYT media with the addition of 100-g/L sucrose (PYTS) was used for the SacB counter-selection. All *Clostridium* strains were incubated at 37°C in an anaerobic cabinet (MG1000 Mark II, 27 Don Whitley, UK; 80% N_2_, 10% CO_2_, and 10% H_2_).

### Plasmids construction

The plasmid details are listed in Table S1, and the details of the primers used are in Table S2. For comparison of tetracycline-inducible systems using GusA assays, two gBlock fragments (COtetR-PIPL12 and COtetR-Pfet) were synthesized by Integrated DNA Technologies with COtetR driven by promoter miniP*4*_tU and tetracycline-inducible promoters P*IPL12* and P*fet* (Table S3). These fragments were ligated into pGG2121 using the Golden Gate assembly (BsaI-HFv2, NEB) along with the *gusA* fragment in pMiniT 2.0-*gusA* to obtain the plasmids pGG-IPL12 and pGG-fet. Moreover, a tetracycline-inducible *gusA* fragment was amplified from plasmid pRPF185 (28) and was ligated into pGG2121 by Golden Gate assembly, resulting in plasmid pGG-RPF185.

The shuttle vector pMTL83221 was obtained from Prof. Minton (SBRC, University of Nottingham) (63). We constructed a new Golden Gate assembling vector, pGG3221, using restriction enzyme-based cloning procedures with vectors pGG2121 and pMTL83221. For the inducible expression of Cas proteins, we digested vector pGG3221 with the BsaI-HFv2 restriction enzyme and purified the 4-kb fragment as the backbone. We then amplified the fragments of *Ascas12a* and *Fncas12a* with BsmBI restriction sites from pDEST-hisMBP-AsCpf1-EC (64) and pY001 (65), respectively. Additionally, we amplified the fragment of COtetR-PIPL12 with BsmBI restriction sites from plasmid pGG-IPL12. We ligated these fragments and the backbone using the Golden Gate assembly protocol (BsmBI-v2, NEB), obtaining the plasmids pP*IPL12*-AsCas12a and pP*IPL12*-FnCas12a.

To construct a series of vectors containing the target and donor DNA templates, we first constructed a general vector. We digested vector pGG2121 with the BsaI-HFv2 restriction enzyme and purified the 4-kb fragment as the backbone. We obtained a fragment of the tetracycline-inducible promoter P*fet* by annealing oligonucleotides. We also obtained fragments containing BsmBI restriction sites and the direct repeat recognized by AsCas12a and FnCas12a (AsDR and FnDR) by annealing oligonucleotides. We ligated these fragments and the backbone using T4 DNA ligase (NEB), obtaining the plasmids pP*fet*-AsDR and pP*fet*-FnDR. We then digested plasmids pP*fet*-AsDR and pP*fet*-FnDR with AscI and BbsI-HF restriction enzymes (NEB) and purified 5-kb fragments as the backbones. Finally, we obtained the fragment containing AatII and SalI restriction sites by annealing oligonucleotides and ligated it into the backbones using T4 DNA ligase, obtaining the general vectors pP*fet*As-Target_v1 and pP*fet*Fn-Target_v1.

To improve plasmid-curing efficiency, we cloned the *sacB* counter-selection marker into pP*fet*Fn-Target_v1 as follows: We digested pP*fet*Fn-Target_v1 with PvuI-HF and PstI-HF restriction enzymes (NEB) and purified a 5-kb fragment as the backbone. Second, the fragment of the *sacB* gene expression cassette was amplified from pGG2121-P*thl*_fU-UUG*sacB* and digested by PvuI and PstI restriction sites. Using T4 DNA ligase (NEB), the fragment and the backbone were ligated, obtaining another general vector, pP*fet*Fn-Target_v2.

The methods for constructing other plasmids based on the general vectors mentioned above can be found in the Supplementary Methods (Text S1). All constructs were verified by Sanger sequencing (GENEWIZ).

### Plasmid transformation

In this study, all plasmids were created in *E. coli* 10-beta and then introduced into *E. coli* S17-1, *gusA* ^−^, and MG1655 RARE through heat shock. The plasmids in *E. coli* S17-1 were then transferred to clostridia using a modified conjugation method (66) in the Supplementary Methods (Text S1).

### Validation of tetracycline-inducible systems by GusA assay

The *E. coli* and *C. butyricum* strains containing a tetracycline-inducible GusA expression plasmid were grown overnight and then sub-cultured into fresh media at a 1:100 dilution. When the OD_600_ of the cultures reached about 0.5, they were split into three volumes. Two of the volumes were induced with 32 and 96 ng/mL of ATc, while the other volume was left non-induced. After 4 hours, 1 mL of each culture was pelleted, and the GusA activity was evaluated as previously described. The GusA activity in mutant variations of the *E. coli* and *C. butyricum* strains was also evaluated, as previously described (38, 54).

### Target selection

To select targets for the Cas12a proteins, we identified regions in the target strand that had Cas12a PAMs (TTTV or TTN). We used CRISPOR (46) and NCBI-BLAST to ensure that the 23-nucleotide target sequence downstream of the 5′ PAM motif was specific to the region. Then, we used RNAfold Web Server (32) to predict the pre-crRNA folding and MFE value.

### Calculation of mutation efficiency

To calculate mutation efficiency, we transformed the plasmids pP*IPL12*-AsCas12a and pP*IPL12*-FnCas12a into clostridia and confirmed trans-conjugants by Sanger sequencing (GENEWIZ). We next transformed the vectors containing the target and the donor DNA template into the clostridia containing either pP*IPL12*-AsCas12a or pP*IPL12*-FnCas12a. Then, at least three trans-conjugants were re-streaked on PYTG agar plates containing D-cycloserine, Tm, Erm, and ATc. Finally, we randomly picked 14 colonies and performed colony PCR. The mutation efficiency was calculated by dividing the number of mutations detected by colony PCR by the total number of colonies in the screen, multiplied by 100. The PCR products of mutations were verified by Sanger sequencing (GENEWIZ).

### Detection of fluorescent intensity

Overnight cultures of *Clostridium* strains were sub-cultured into fresh PYTG media (2:100) with substrates added. For each time point, 1 mL of the culture was washed once with PBS by centrifugation and suspended in 1 mL of PBS. The fluorescent intensity of 100 µL of the suspension was measured on a 96-well polystyrene plate (P8741, Sigma) using a SpectraMax iD3 Multi-Mode Microplate Reader with excitation and emission wavelengths of 660 and 730 nm, respectively. The rest of the suspension was used to measure the OD_600_.

### Confocal imaging

To prepare samples for confocal imaging, *Clostridium* strains were sub-cultured into fresh PYTG media at a 1:100 dilution and grown with substrates for 6 hours. After washing the cells once in PBS, they were fixed in 4% formaldehyde (28,906, Thermo Scientific) in the dark for 15 minutes, followed by two additional washes in PBS. The fixed cells were mixed with an equal volume of fluorescent mounting medium (S3023, Dako) and mounted on a glass slide with a cover slip for overnight incubation. The confocal images of the cells were captured using a Leica SPE confocal laser scanning microscope (Leica Microsystems GmbH) equipped with diode lasers of 488 and 635 nm and oil immersion objectives (×63, numerical aperture = 1.4) (67). Three scans were performed for each image, and the images were analyzed and exported using Leica Application Suite X (LAS X).

## Data Availability

All plasmids used in this study are listed in Table S1 and are available upon request. The entry vectors for the CRISPR-FnCas12a system were deposited to Addgene as accession numbers #199563 for pPIPL12-FnCas12a and #199564 for pPfetFn-Target_v2.

## SUPPLEMENTAL MATERIAL

Supplemental Material spectrum.02459-23-s0001.pdf Figures S1 to S8, Tables S1 to S3, Text S1.

ASM does not own the copyrights to Supplemental Material that may be linked to, or accessed through, an article. The authors have granted ASM a non-exclusive, world-wide license to publish the Supplemental Material files. Please contact the corresponding author directly for reuse.
