# Supplementary material for: Development of a CRISPR-Cas12a system for efficient genome engineering in clostridia
Source: Microbiol Spectr. 2023 Nov 10;11(6):e02459-23. doi: 10.1128/spectrum.02459-23 (PMC10715149; doi:10.1128/spectrum.02459-23)
Supplement: Supplemental Material — Figures S1 to S8, Tables S1 to S3, Text S1. [file spectrum.02459-23-s0001.pdf]

Supplemental Material

Development of a CRISPR-Cas12a System for Efficient Genome Engineering in Clostridia

Yanchao Zhang[1,*], Aleksandra M. Kubiak[1,2], Tom S. Bailey[1], Luuk Claessen[1,3], Philip Hittmeyer[1,3], Ludwig Dubois[1], Jan Theys[1], Philippe Lambin[1,*]

[1] The M-Lab, Department of Precision Medicine, GROW - School of Oncology and Reproduction, Maastricht University, 6229 ER Maastricht, the Netherlands

[2] Exomnis Biotech BV, Oxfordlaan 55, 6229 EV Maastricht, the Netherlands

[3] LivingMed Biotech BV, Clos Chanmurly 13, 4000 Liège, Belgium

* To whom correspondence should be addressed.

Email: yanchao.zhang@maastrichtuniversity.nl & philippe.lambin@maastrichtuniversity.nl

Figure S1. Optimized tetracycline-inducible gene expression systems in *E. coli* and *C. butyricum*
Figure S2. The prediction of the folding of Pre-crRNAs in the CRISPR-AsCas12a system by the RNAfold Web Server
Figure S3. The *uidA* gene deletion in *E. coli* MG1655 RARE by the CRISPR-FnCas12a system
Figure S4. Plasmid curing
Figure S5. Characterization of the mutation phenotypes
Figure S6. Growth and NIR emission of genetically engineered clostridia in the presence of hemin
Figure S7. Confocal imaging of the CB_WT, the CS_WT, and *C. butyricum* expressing UnaG, CreiLOV, and IFP2.0
Figure S8. Integration of the hemin biosynthetic pathway into *C. sporogenes*
Table S1. Strains and plasmids used in this work
Table S2. Primers used in the study
Table S3. Sequences of gBlock fragments, codon-optimized and synthesized by Integrated DNA Technologies
Text S1. Supplementary methods

Figure S1. Optimized tetracycline-inducible gene expression systems in *E. coli* and *C. butyricum*. (A) The schema and sequences of optimized tetracycline-inducible systems. Compared to the control RPF185, the optimizations were highlighted in red. The sequences of the Tet operator and ribosome-binding site were highlighted in bold and underlined, respectively. *COtetR*, the codon-optimized Tet repressor *tetR*. Validation of tetracycline-inducible systems using the glucuronidase assay in *E. coli* (B) and *C. butyricum* (C). The data represent the mean ± s.d. of three biological replicates.

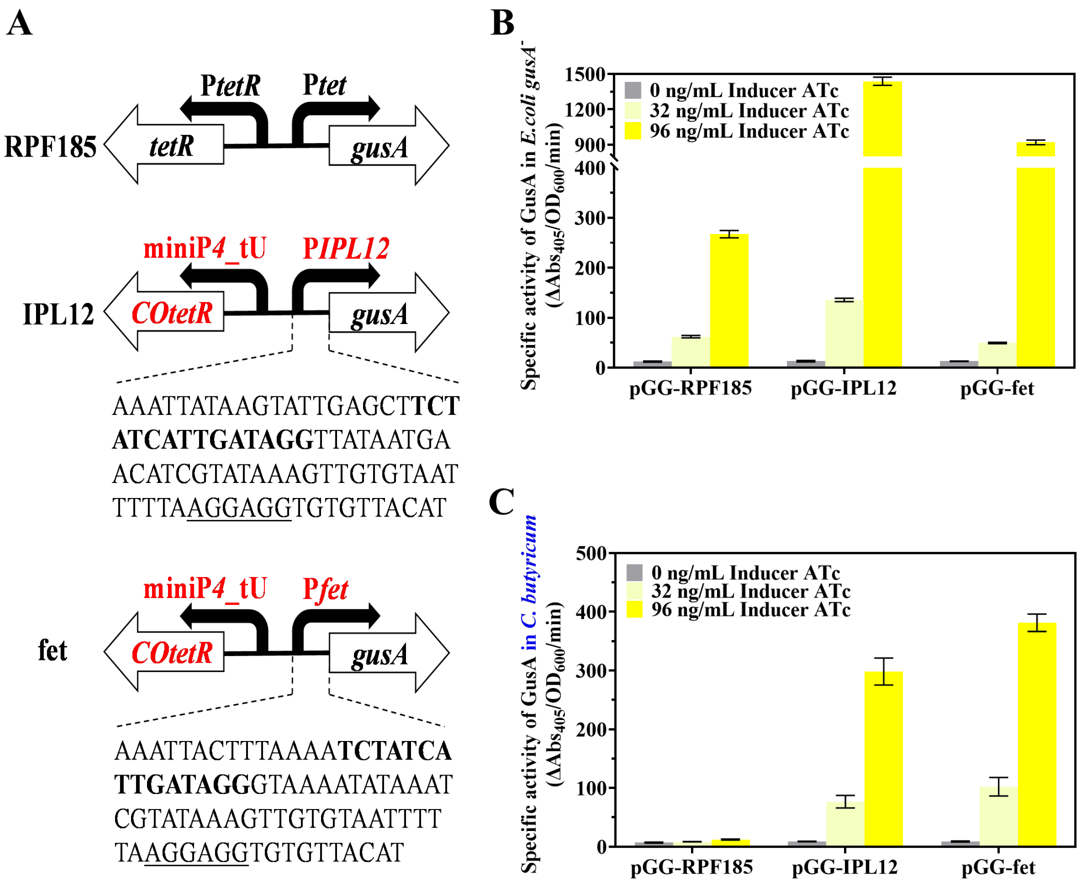

Figure S2. Prediction of the folding of Pre-crRNAs in the CRISPR-AsCas12a system by the RNAfold Web Server. Target sequences of (A) 5’-catcaacatctccttggctcttt-3’, (B) 5’-gcttttccaacacccctaaacca-3’, (C) 5’-aaacttgtgcaatagtaactatg-3’, and (D) 5’-atagtgctccaacaatacaagaa-3’.

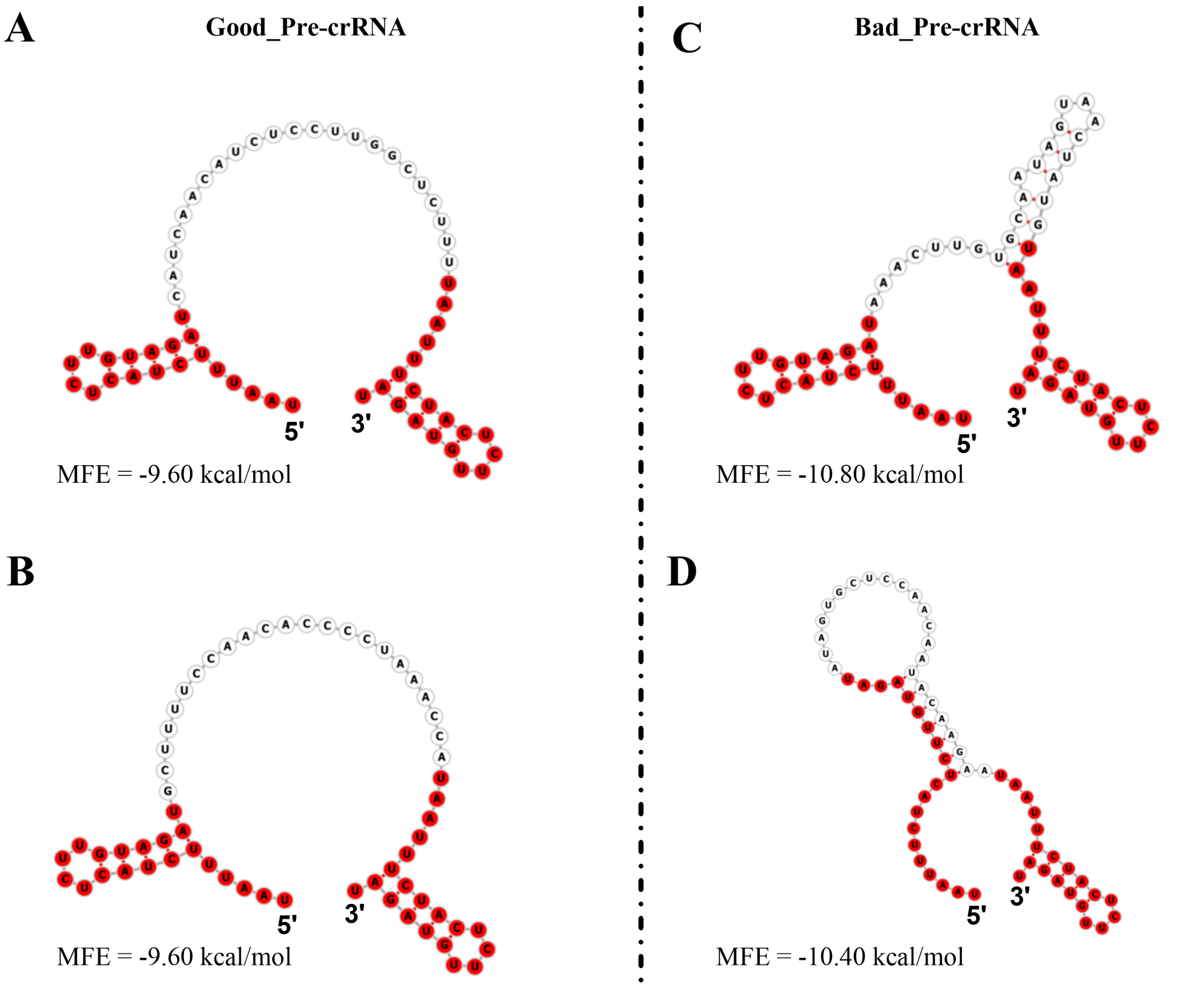

Figure S3. The *uidA* gene deletion in *E. coli* MG1655 RARE by the CRISPR-FnCas12a system. The recognized PAMs of the target sequences were highlighted in red. The PCR products of the *uidA* mutation and the wild type were 1931 bp and 3055 bp, respectively. Quick-Load Purple 1 kb Plus DNA (N0550L, NEB) was used as the molecular weight standard. 1–14, PCR products from single colonies; W, PCR products from the wild-type genome.

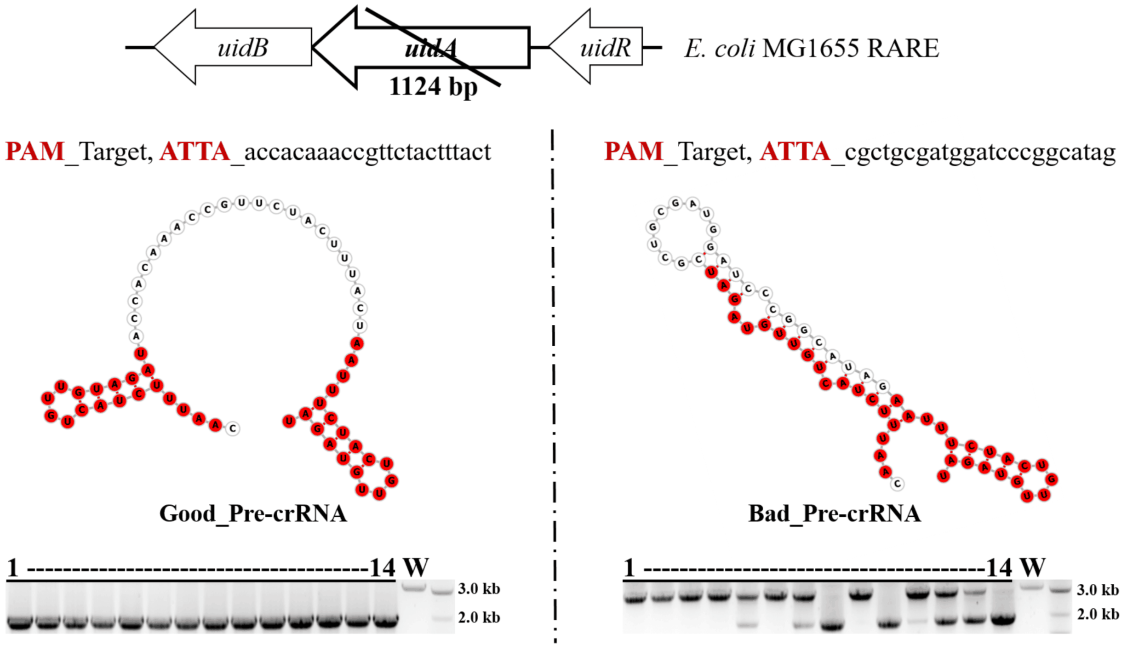

Figure S4. Plasmid curing in (A) *C. butyricum*, (B) *C. sporogenes*, and (C) *E. coli* MG1655 RARE. Tm, thiamphenicol; Erm, erythromycin; Cm, chloramphenicol.

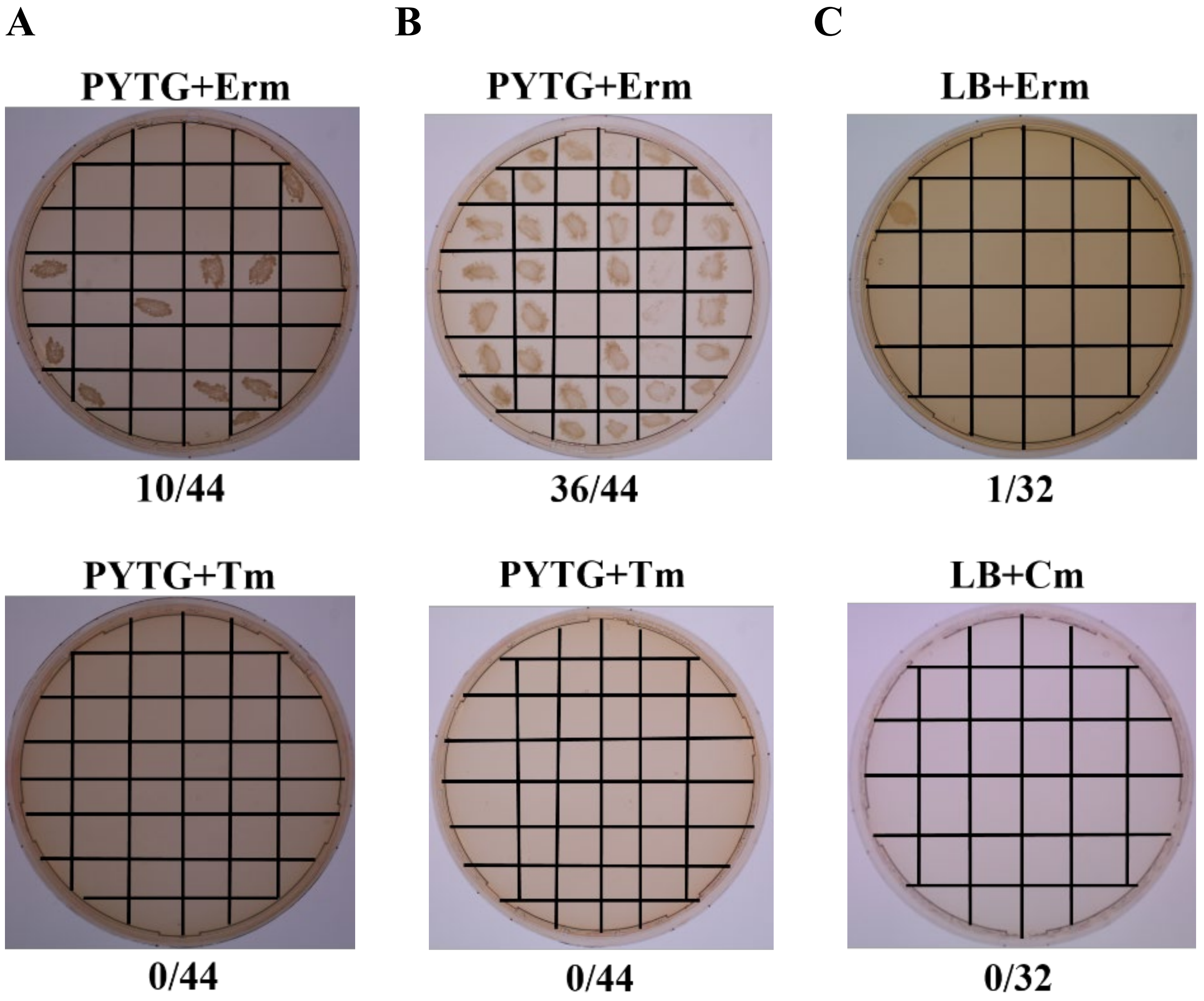

Figure S5. Characterization of the mutation phenotypes. (A) The CB_WT and CB_Δ*pyrE* strains on PYTG agar plates with or without the addition of 1 mg/mL 5-FOA. (B) Sporulation assay of the CB_WT and CB_Δ*spo0A* strains. (C) Glucuronidase activities of the CB_WT, CB_IN*gusA*, RARE_WT, and RARE_Δ*uidA* strains. The data represent the mean ± s.d. of three biological replicates.

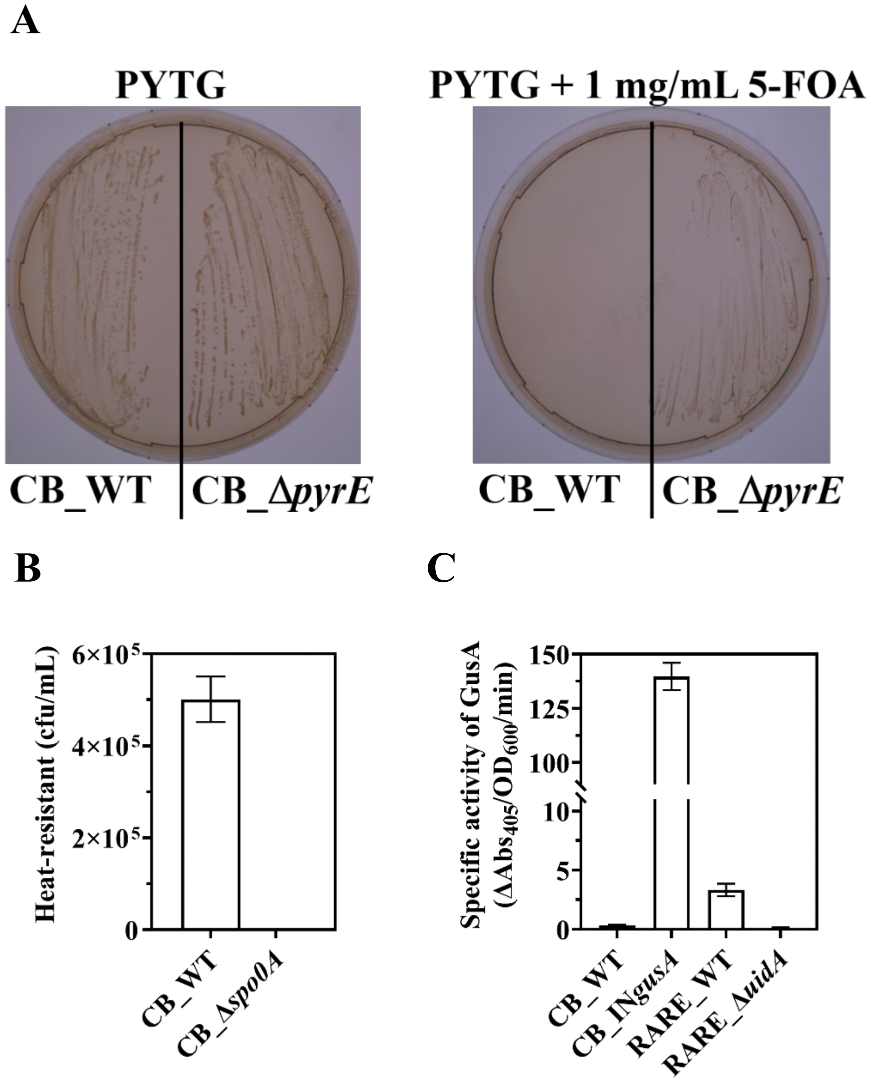

Figure S6. Growth and NIR emission of genetically engineered clostridia in the presence of hemin. Growth of strains CB003 (A) and CS003 (C) and intensity of near-infrared fluorescence (A.U. in 660/730 nm of Ex/Em) over time in strains CB003 (B) and CS003 (D) was measured in the presence of different hemin concentrations. (E) Colony PCR to confirm integration of the HemO expression cassette with promoter P*thl*_fU in the CB001 and CS001 strains, obtaining CB002 (size of mutation/wild type, 3136/2338 bp) and CS002 (size of mutation/wild type, 3122/2343 bp) strains. GeneRuler 1 kb DNA ladder (SM0311, Thermo Scientific) was used as the molecular weight standard. 1–14, PCR products from single colonies; W, PCR products from the wild-type genome. Growth of in CB002 (F) and CS002 (H) strains and the intensity of near-infrared fluorescence (A.U. in 660/730 nm of Ex/Em) over time in CB002 (G) and CS002 (I) was measured in the presence of different hemin concentrations. The data represent the mean ± s.d. of three biological replicates.

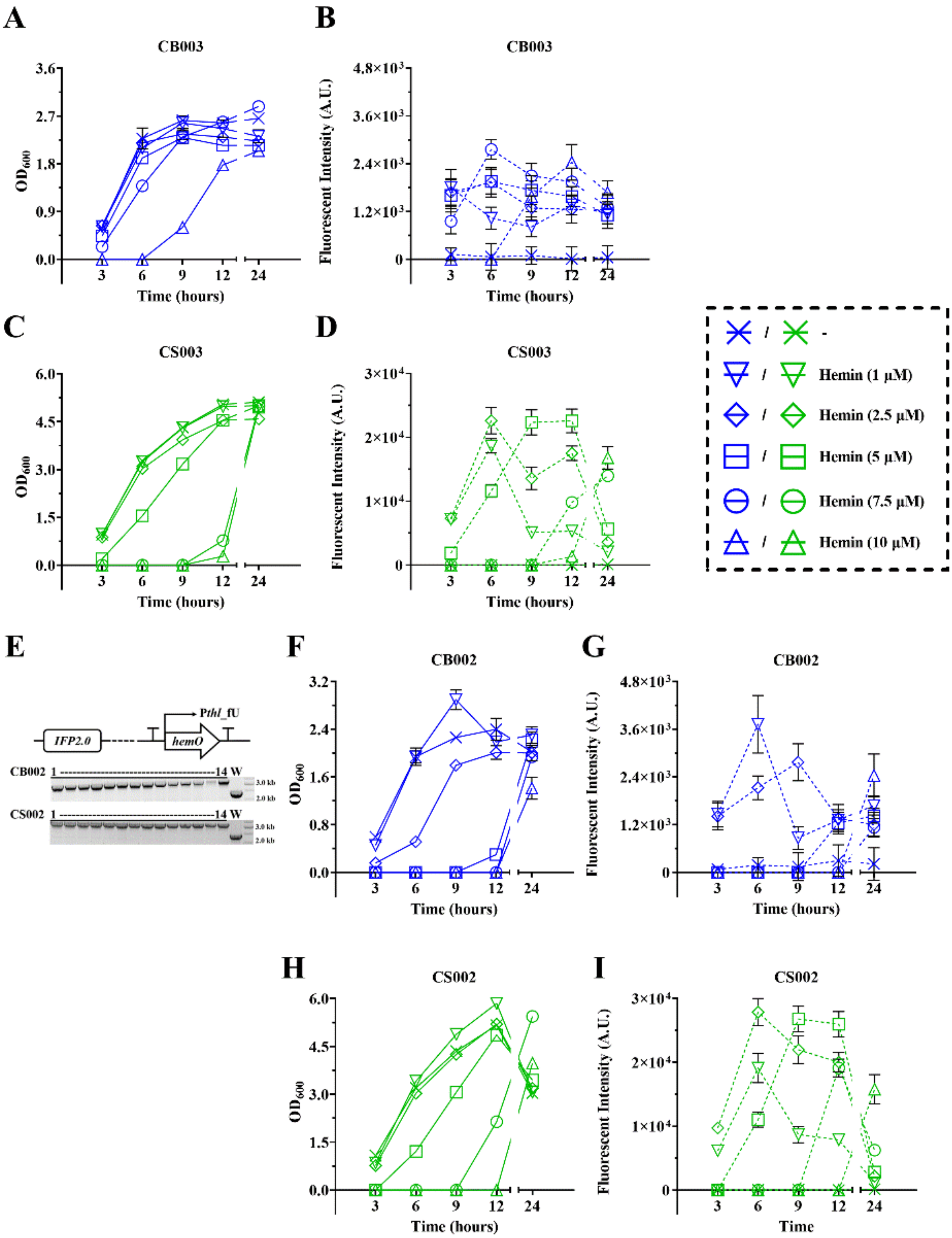
A
CB003
OD600
Time (hours)
B
CB003
Fluorescent Intensity (A.U.)
Time (hours)
C
CS003
OD600
Time (hours)
D
CS003
Fluorescent Intensity (A.U.)
Time (hours)
/ -
/ Hemin (1 μM)
/ Hemin (2.5 μM)
/ Hemin (5 μM)
/ Hemin (7.5 μM)
/ Hemin (10 μM)
E
Pthl_fU
IFP2.0
hemO
CB002 1 14 W
CS002 1 14 W
F
CB002
OD600
Time (hours)
G
CB002
Fluorescent Intensity (A.U.)
Time (hours)
H
CS002
OD600
Time (hours)
I
CS002
Fluorescent Intensity (A.U.)
Time

Figure S7. Confocal imaging of the CB_WT, the CS_WT, and *C. butyricum* expressing UnaG, CreiLOV, and IFP2.0. CB_WT (A) and CS_WT (B) in the presence of 10 μM Bv and 7.5 μM hemin at 488 nm, 635 nm, and merge channels. *C. butyricum* containing pGG2121 in the presence of 25 μM bilirubin (Br), 25 μM riboflavin 5′-monophosphate sodium salt (FMN), and 25 μM Bv (C). *C. butyricum* containing pGG2121-P*fdx-UnaG* without and with the addition of 25 μM Br at 488nm (D). *C. butyricum* containing pGG2121-P*fdx-CreiLOV* without and with the addition of 25 μM FMN at 488nm (E). *C. butyricum* containing pGG2121-P*fdx-IFP2.0* without and with the addition of 25 μM Bv at 635nm (F).

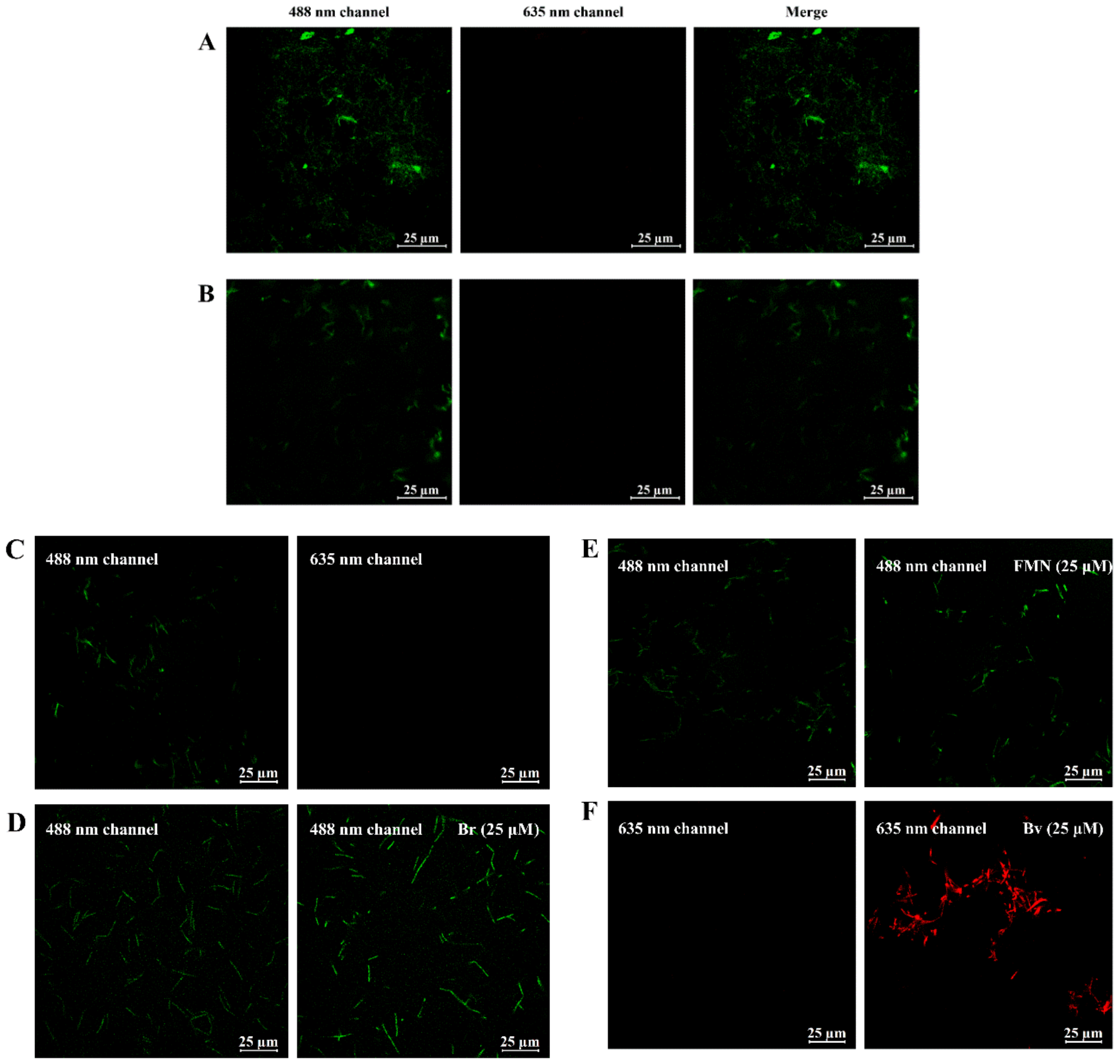

Figure S8. Integration of the hemin biosynthetic pathway into *C. sporogenes*. (A) The metabolic genes of hemin biosynthesis from *C. butyricum* (blue), *C. sporogenes* (black), and *E. coli* (red). GtrR, glutamyl-tRNA reductase; GSAM, glutamate-1-semialdehyde aminomutase; PBGS, porphobilinogen synthase; PBGD, porphobilinogen deaminase; UROS, uroporphyrinogen III synthase; UROD, uroporphyrinogen III decarboxylase; CPO, coproporphyrinogen III oxidase; PPO, protoporphyrinogen oxidase; FECH, ferrochelatase; α-KG, α-ketoglutarate; GSA, glutamate-1-semialdehyde; ALA, δ-aminolevulinic acid; PBG, porphobilinogen; HMB, hydroxymethylbilane. (B) The schema and colony PCR of integrating multiple polycistronic expression cassettes (*hemLA*, *hemNG*, and *hemEH*) into the CS003 strain to obtain the final CS006 strain (size of mutation/wild type, 5447/2787 bp, 5716/3775 bp, and 5511/3237 bp, respectively). With the addition of 12.5 mg/L menaquinone-4 and 20 mg/L ferrous sulfate (*), 10 µM Bv, or 2.5 µM hemin, the growth curve and the intensity of near-infrared fluorescence (A.U. in 660/730 nm of Ex/Em) in CS_WT (C) and CS006 (D) strains.

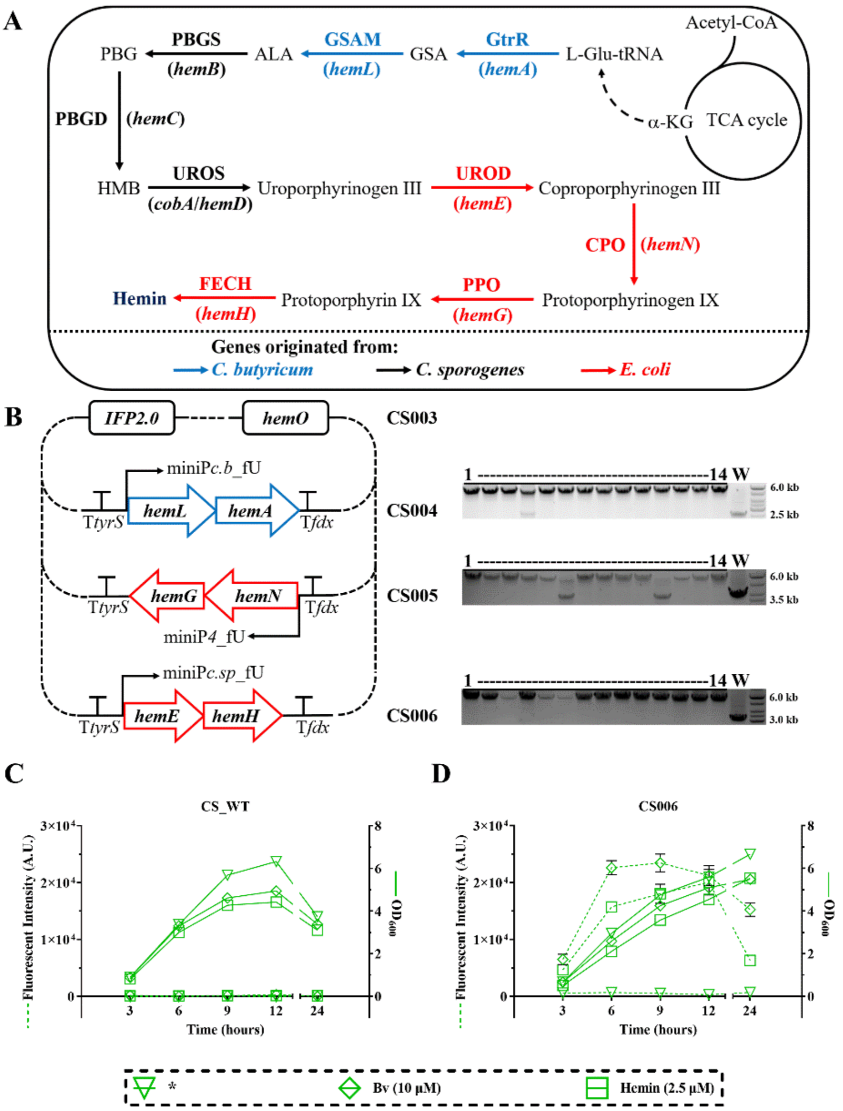

Table S1. Strains and plasmids used in this work

Strains	Description	Sources
E. coli 10-beta	High-efficiency strain ideal for cloning	C3019, NEB
E. coli gusA$^-$	*E. coli* JW1609 with the *gusA* knockout mutant	OEC4987-200827007, Horizon Discovery Ltd.
E. coli S17-1	Conjugative donor strain	ATCC 47055
E. coli MG1655 RARE/ RARE-WT	The following genes were deleted from the *E. coli* K-12 MG1655 genome: *dkgB*, *yeaE*, *dkgA*, *yqhC*, *yqhD*, *yjgB*, and *yahK*.	61440, Addgene
RARE_Δ*uidA*	*E. coli* MG1655 RARE harboring deletion of the *uidA* gene	This work
C. sporogenes/CS_WT	Wild type of *Clostridium sporogenes* NCIMB 10696	NCIMB culture collection (UK)
C. butyricum/CB_WT	Wild type of *Clostridium butyricum* DSM10702	DSMZ-German Collection
CB_Δ*pyrE*	*C. butyricum* harboring deletion of the *pyrE* gene	This work
CB_Δ*spo0A*	*C. butyricum* harboring deletion of the *spo0A* gene	This work
CB_IN*gusA*	*C. butyricum* harboring integration of the *gusA* expression cassette	This work
CB001	CB_WT harboring integration of the *IFP2.0* expression cassette	This work
CB002	CB001 harboring integration of the *hemO* expression cassette with promoter P*thl*_fU	This work
CB003	CB001 harboring integration of the *hemO* expression cassette with promoter P*thl*79%-26_fU	This work
CS001	CS_WT harboring integration of the *IFP2.0* expression cassette	This work
CS002	CS001 harboring integration of the *hemO* expression cassette with promoter P*thl*_fU	This work
CS003	CS001 harboring integration of the *hemO* expression cassette with promoter P*thl*79%-26_fU	This work
CS004	CS003 harboring integration of the *hemL-hemA* polycistronic expression cassette	This work
CS005	CS004 harboring integration of the *hemN-hemG* polycistronic expression cassette	This work
CS006	CS005 harboring integration of the *hemE-hemH* polycistronic expression cassette	This work

Number	Plasmids	Description	Sources
	pGG2121	Golden Gate assembling vector based on pMTL82121, Cm^r, Gram$^-$ replicon p15a, Gram$^+$ replicon pBP1	(1)
	pMTL3221	*E. coli* - *Clostridium* shuttle vector, Erm^r, Gram$^-$ replicon p15a, Gram positive replicon pCB102	(2)
	pGG3221	Golden Gate assembling vector based on pGG2121 and pMTL3221, Erm^r, Gram$^-$ replicon p15a, Gram positive replicon pCB102	This work
	pGG2121-P*thl*_fU-UUG*sacB*	pGG2121 ligating with the P*thl*_fU and the *sacB* gene with UUG start codon	(1)
	pGG2121-P*romoter*-*gusA*	pGG2121 ligating with promoters and the *gusA* gene	
	pMiniT 2.0-*gusA*	The *gusA* fragment with Golden Gate *Bsa*I sites in pMiniT 2.0	(1)
	pRPF185	*E. coli* - *C. difficile* shuttle vector containing the codon optimized gene *gusA* for *C. difficile*	106367, Addgene

	pDEST-hisMBP-AsCpf1-EC	A gift from Jin-Soo Kim, expressing his-MBP tagged AsCpf1	79007, Addgene
	pY001	A gift from Feng Zhang, expresses FnCpf1 locus	69973, Addgene
	pGG-RPF185	pGG2121 ligating with the tetracycline-inducible *gusA* from pRPF185	This work
	pGG-IPL12	pGG2121 ligating with the *gusA* gene driven by the COtetR-PIPL12	This work
	pGG-fet	pGG2121 ligating with the *gusA* gene driven by the COtetR-Pfet	This work
	pP*IPL12*-AsCas12a	pGG3221 ligating with the *Ascas12a* gene driven by the COtetR-PIPL12, Erm^{r}	This work
	pP*IPL12*-FnCas12a	pGG3221 ligating with the *Fncas12a* gene driven by the COtetR-PIPL12, Erm^{r}	This work
	pP*fet*-AsDR	pGG2121 ligating with the Direct Repeat of AsCas12a driven by the P*fet*	This work
	pP*fet*-FnDR	pGG2121 ligating with the Direct Repeat of FnCas12a driven by the P*fet*	This work
	pP*fet*As-Target_v1	pP*fet*-AsDR ligating with the fragment containing AatII and SalI restriction sites, Cm^{r}	This work
	pP*fet*Fn-Target_v1	pP*fet*-FnDR ligating with the fragment containing AatII and SalI restriction sites, Cm^{r}	This work
V1-1	pP*fet*As-D*spo0A*-Tn_v1	pP*fet*As-Target_v1 ligating with the donor DNA template and different targets for deleting gene *spo0A* in *C. butyricum*	This work
V1-2	pP*fet*As-D*pyrE*-Tn_v1	pP*fet*As-Target_v1 ligating with the donor DNA template and different targets for deleting gene *pyrE* in *C. butyricum*	This work
V1-3	pP*fet*Fn-D*spo0A*-Tn_v1	pP*fet*Fn-Target_v1 ligating with the donor DNA template and different targets for deleting gene *spo0A* in *C. butyricum*	This work
V1-4	pP*fet*Fn-D*pyrE*-Tn_v1	pP*fet*Fn-Target_v1 ligating with the donor DNA template and different targets for deleting gene *pyrE* in *C. butyricum*	This work
V1-5	pP*fet*Fn-D*uidA*-Tn_v1	pP*fet*Fn-Target_v1 ligating with the donor DNA template and different targets for deleting gene *uidA* in *E. coli* MG1655 RARE	This work
	pP*fet*Fn-Target_v2	pP*fet*Fn-Target_v1 ligating with the *sacB* gene expression cassette, Cm^{r}	This work
V2-3	pP*fet*Fn-D*spo0A*-Tn_v2	pP*fet*Fn-Target_v2 ligating with the donor DNA template and different targets for deleting gene *spo0A* in *C. butyricum*	This work
V2-4	pP*fet*Fn-D*pyrE*-Tn_v2	pP*fet*Fn-Target_v2 ligating with the donor DNA template and different targets for deleting gene *pyrE* in *C. butyricum*	This work
V2-5	pP*fet*Fn-D*uidA*-Tn_v2	pP*fet*Fn-D*uidA*-Tn_v1 ligating with the *sacB* gene expression cassette	This work
V2-6	pP*fet*Fn-Inc.bGusA_1.5k-Tn	pP*fet*Fn-Target_v2 ligating with the donor DNA template of 1.5k donor arms and different targets for integrating the GusA expression cassette in *C. butyricum*	This work
V2-7	pP*fet*Fn-Inc.bGusA_1.0k-Tn	pP*fet*Fn-Target_v2 ligating with the donor DNA template of 1.0k donor arms and different targets for integrating the GusA expression cassette in *C. butyricum*	This work
V2-8	pP*fet*Fn-Inc.bGusA_0.75k-Tn	pP*fet*Fn-Target_v2 ligating with the donor DNA template of 0.75k donor arms and different targets for integrating the GusA expression cassette in *C. butyricum*	This work

V2-9	pP*fet*Fn-Inc.bGusA_0.5k-Tn	pP*fet*Fn-Target_v2 ligating with the donor DNA template of 0.5k donor arms and different targets for integrating the GusA expression cassette in *C. butyricum*	This work
V2-10	pP*fet*Fn-Inc.bGusA_0.25k-Tn	pP*fet*Fn-Target_v2 ligating with the donor DNA template of 0.25k donor arms and different targets for integrating the GusA expression cassette in *C. butyricum*	This work
P1	pGG2121-P*fdx-IFP2.0*	pGG2121 ligating with the P*fdx* and the *IFP2.0* gene	This work
P2	pGG2121-P*fdx-UnaG*	pGG2121 ligating with the P*fdx* and the *UnaG* gene	This work
P3	pGG2121-P*fdx-CreiLOV*	pGG2121 ligating with the P*fdx* and the *CreiLOV* gene	This work
V2-11	pP*fet*Fn-Inc.bIFPUD-T1	pP*fet*Fn-Target_v2 ligating with the donor DNA template and target for integrating the IFP2.0 expression cassette in *C. butyricum*	This work
V2-12	pP*fet*Fn-InspIFPUD-T1	pP*fet*Fn-Target_v2 ligating with the donor DNA template and target for integrating the IFP2.0 expression cassette in *C. sporogenes*	This work
P4	pGG2121-P*thl*_fU-*hemO*	pGG2121 ligating with the P*thl*_fU and the *hemO* gene	This work
V2-13	pP*fet*Fn-Inc.bHemOUD-T1	pP*fet*Fn-Target_v2 ligating with the donor DNA template and target for integrating the HemO expression cassette with promoter P*thl*_fU in *C. butyricum*	This work
V2-14	pP*fet*Fn-InspHemOUD-T1	pP*fet*Fn-Target_v2 ligating with the donor DNA template and target for integrating the HemO expression cassette with promoter P*thl*_fU in *C. sporogenes*	This work
P5	pGG2121-P*thl*79%-26_fU-*hemO*	pGG2121 ligating with the P*thl*79%-26_fU and the *hemO* gene	This work
V2-15	pP*fet*Fn-Inc.b7926HemOUD-T1	pP*fet*Fn-Target_v2 ligating with the donor DNA template and target for integrating the HemO expression cassette with promoter P*thl*79%-26_fU in *C. butyricum*	This work
V2-16	pP*fet*Fn-Insp7926HemOUD-T1	pP*fet*Fn-Target_v2 ligating with the donor DNA template and target for integrating the HemO expression cassette with promoter P*thl*79%-26_fU in *C. sporogenes*	This work
P6	pGG2121-miniP*c.b*_fU-*hemLA*	pGG2121 ligating with the miniP*c.b*_fU and the *hemLA* polycistronic gene	This work
V2-17	pP*fet*Fn-InspHemLAUD-T1	pP*fet*Fn-Target_v2 ligating with the donor DNA template and target for integrating the *hemLA* polycistronic expression cassette in *C. sporogenes*	This work
P7	pGG2121-miniP*4*_fU-*hemNG*	pGG2121 ligating with the miniP*4*_fU and the *hemNG* polycistronic gene	This work
V2-18	pP*fet*Fn-InspHemNGUD-T1	pP*fet*Fn-Target_v2 ligating with the donor DNA template and target for integrating the *hemNG* polycistronic expression cassette in *C. sporogenes*	This work
P8	pGG2121-miniP*c.sp*_fU-*hemEH*	pGG2121 ligating with the miniP*c.sp*_fU and the *hemEH* polycistronic gene	This work
V2-19	pP*fet*Fn-InspHemEHUD-T1	pP*fet*Fn-Target_v2 ligating with the donor DNA template and target for integrating the *hemEH* polycistronic expression cassette in *C. sporogenes*	This work

Table S2. Primers used in the study. Restriction sites are underlined.

Number	Primers	5' - 3' Sequences	Description
1	TetR-gusA-F	GGTCTCGTCCATTAAGACCCACTTTCACATTTAAG	Amplifying the fragment of tetracycline-inducible *gusA* from pRPF185
2	BsaI-gusA-R	TATAGGTCTCAGTCTTCATTGTTTGCCTCCCTGC	
3	PIPL12-BsmBI-F	CGTCTCGTCCATTAAGACCCACTTTCACATTTAA	Amplifying the fragment of COtetR-PIPL12 from plasmid pGG-IPL12
4	PIPL12-BsmBI-R	CGTCTCTATGTAACACACCTCCTTAAAAAT	
5	AsCas12a-BsmBI-F	CGTCTCGACATATGACACAGTTTGAAGGCTTC	Amplifying the fragment of *Ascas12a* from pDEST-hisMBP-AsCpf1-EC
6	AsCas12a-BsmBI-R	CGTCTCGGTCTGTTTCTCAGTTCTTGAATGTAGG	
7	FnCas12a-BsmBI-F	CGTCTCGACATATGTCAATTTATCAAGAATTTGTTAATAAATATAG	Amplifying the fragment of *Fncas12a* from pY001
8	FnCas12a-BsmBI-R	CGTCTCGGTCTTTAGTTATTCCTATTCTGCACG	
9	Pfet-oligo-F	TCCAAAATTACTTTAAAATCTATCATTGATAGGGTAAAATATA	Oligonucleotides annealing for the fragment of P*fet*
10	Pfet-oligo-R	GATTTATATTTTACCCTATCAATGATAGATTTTAAAGTAATTT	
11	FnDR-oligo-F	AATCAATTTCTACTGTTGTAGATTGAGACGATTCGTCTCCAATTTCTACTGTTGTAGATTTTTTT	Oligonucleotides annealing for the fragment of FnDR
12	FnDR-oligo-R	GTCTAAAAAAATCTACAACAGTAGAAATTGGAGACGAATCGTCTCAATCTACAACAGTAGAAATT	
13	AsDR-oligo-F	AATCTAATTTCTACTCTTGTAGATTGAGACGATTCGTCTCCTAATTTCTACTCTTGTAGATTTTTTT	Oligonucleotides annealing for the fragment of AsDR
14	AsDR-oligo-R	GTCTAAAAAAATCTACAAGAGTAGAAATTAGGAGACGAATCGTCTCAATCTACAAGAGTAGAAATTA	
15	AatII-SalI-oligo-F	CGCGACGTCGTTCTGAATCCTTAGCTAATGGGTCGAC	Oligonucleotides annealing for the fragment containing AatII and SalI restriction sites
16	AatII-SalI-oligo-R	ATACGTCGACCCATTAGCTAAGGATTCAGAACGACGT	
17	SacB-PvuI-F	GCCGATCGTTTTTAACAAAATATATTGATAAAAATAATAATAGTGGGT	Amplifying the fragment of the *sacB* gene expression cassette from pGG2121-P*thl*_fU-UUG*sacB*
18	SacB-PstI-R	CGCTGCAGTTATTTGTTAACTGTTAATTGTCCTTG	
19	c.bDspo0A-U-AatII-F	GAGAGGACGTCCGGTCAATTAAACAGTAATATACTTC	Amplifying the fragment upstream for deleting gene *spo0A* from the genome of *C. butyricum* DSM10702
20	c.bDspo0A-U-BsmBI-R	CGTCTCCGCTCCTATTAATGCACTTTTTATATGTTTTCC	
21	c.bDspo0A-D-BsmBI-F	CGTCTCGGAGCTATTTTATTGGGCTGAG	Amplifying the fragment downstream for deleting gene *spo0A* from the genome of *C. butyricum* DSM10702
22	c.bDspo0A-D-SalI-R	GCGCGTCGACAAGTACCTTTCTTTGTAATCAATCC	
23	c.bDspo0A-F	AGGAGAAAAAGGTTCTCCTGGAGAA	Colony PCR for deleting gene *spo0A* from the genome of *C. butyricum* DSM10702
24	c.bDspo0A-R	CTCCTGACAGCAAGAAACGCC	

25	c.bDpyrE-U-AatII-F	GAGAGGACGTCGATGTAGGAATTCTTGGAGG	Amplifying the fragment upstream for deleting gene *pyrE* from the genome of *C. butyricum* DSM10702
26	c.bDpyrE-U-BsmBI-R	CGTCTCCTGTAGTTAACTTCAAACTTTTTATTAAATAATTCC	
27	c.bDpyrE-D-BsmBI-F	CGTCTCGTACAATATATAGGGAAAAATAAGGTGAT	Amplifying the fragment downstream for deleting gene *pyrE* from the genome of *C. butyricum* DSM10702
28	c.bDpyrE-D-SalI-R	GCGCGTCGACAGTCACTTACTTCATCCCATAAC	
29	c.bDpyrE-F	TGCTGTGGCCCTGAAGTTATGA	Colony PCR for deleting gene *pyrE* from the genome of *C. butyricum* DSM10702
30	c.bDpyrE-R	ACTTCATATCCATAAGCCTTAGCTGCA	
31	c.bDspo0A-TTTA_Good-BsmBI-F	CGTCTCGAGATGCTTTTCCAACACCCCTAAAC	For deleting gene *spo0A* in *C. butyricum* by AsCas12a or FnCas12a, amplifying the fragments of the target for Good_Pre-crRNA recognizing 5'-TTTA PAM
32	c.bDspo0A-As_TTTA_Good-BsmBI-R	CGTCTCGATTATGGTTTAGGGGTGTTGGAAAAG	
33	c.bDspo0A-Fn_TTTA_Good-BsmBI-R	CGTCTCGAATTTGGTTTAGGGGTGTTGGAAAAG	
34	c.bDspo0A-TTTA_Bad-BsmBI-F	CGTCTCGAGATATAGTGCTCCAACAATACAAGAA	For deleting gene *spo0A* in *C. butyricum* by AsCas12a or FnCas12a, amplifying the fragment of the target for Bad_Pre-crRNA recognizing 5'-TTTA PAM
35	c.bDspo0A-As_TTTA_Bad-BsmBI-R	CGTCTCGATTATTCTTGTATTGTTGGAGCAC	
36	c.bDspo0A-Fn_TTTA_Bad-BsmBI-R	CGTCTCGAATTTTCTTGTATTGTTGGAGCAC	
37	c.bDpyrE-TTTA_Good-BsmBI-F	CGTCTCGAGATCATCAACATCTCCTTGGCTCT	For deleting gene *pyrE* in *C. butyricum* by AsCas12a or FnCas12a, amplifying the fragments of the target for Good_Pre-crRNA recognizing 5'-TTTA PAM
38	c.bDpyrE-As_TTTA_Good-BsmBI-R	CGTCTCGATTAAAAGAGCCAAGGAGATGTTG	
39	c.bDpyrE-Fn_TTTA_Good-BsmBI-R	CGTCTCGAATTAAAGAGCCAAGGAGATGTTG	
40	c.bDpyrE-TTTA_Bad-BsmBI-F	CGTCTCGAGATAAACTTGTGCAATAGTAACTATG	For deleting gene *pyrE* in *C. butyricum* by AsCas12a or FnCas12a, amplifying the fragment of the target for Bad_Pre-crRNA recognizing 5'-TTTA PAM
41	c.bDpyrE-As_TTTA_Bad-BsmBI-R	CGTCTCGATTACATAGTTACTATTGCACAAGTTTATCT	
42	c.bDpyrE-Fn_TTTA_Bad-BsmBI-R	CGTCTCGAATTCATAGTTACTATTGCACAAGTTTATCT	
43	c.bDpyrE-CTTA_Good-BsmBI-F	CGTCTCGAGATTCTCCATCATTTATCTTACTTCC	For deleting gene *pyrE* in *C. butyricum* by AsCas12a or FnCas12a, amplifying the fragments of the target for Good_Pre-crRNA recognizing 5'-CTTA PAM
44	c.bDpyrE-As_CTTA_Good-BsmBI-R	CGTCTCGATTAGGAAGTAAGATAAATGATGGAGAA	
45	c.bDpyrE-Fn_CTTA_Good-BsmBI-R	CGTCTCGAATTGGAAGTAAGATAAATGATGGAGAA	
46	c.bDpyrE-CTTA_Bad-BsmBI-F	CGTCTCGAGATCTTCCTAACAAGATCCCTTTATCA	For deleting gene *pyrE* in *C. butyricum* by FnCas12a, amplifying the fragment of the target for Bad_Pre-crRNA recognizing 5'-CTTA PAM
47	c.bDpyrE-Fn_CTTA_Bad-BsmBI-R	CGTCTCGAATTGATAAAGGGATCTTGTTAGGAAGATCT	

48	c.bDpyrE-ATTA_Good-BsmBI-F	CGTCTCGAGATTAAGCCCTTTTACATCAACATCT	For deleting gene *pyrE* in *C. butyricum* by AsCas12a or FnCas12a, amplifying the fragment of the target for Good_Pre-crRNA recognizing 5’-ATTA PAM
49	c.bDpyrE-As_ATTA_Good-BsmBI-R	CGTCTCGATTAAGATGTTGATGTAAAAGGGCTTAATCT	
50	c.bDpyrE-Fn_ATTA_Good-BsmBI-R	CGTCTCGAATTAGATGTTGATGTAAAAGGGCTTAATCT	
51	c.bDpyrE-ATTA_Bad-BsmBI-F	CGTCTCGAGATGTTGAAATAAGAGAAAAATTTGGAATT	For deleting gene *pyrE* in *C. butyricum* by FnCas12a, amplifying the fragment of the target for Bad_Pre-crRNA recognizing 5’-ATTA PAM
52	c.bDpyrE-Fn_ATTA_Bad-BsmBI-R	CGTCTCGAATTCCAAATTTTTCTCTTATTTCAACATCTC	
53	c.bDpyrE-GTTA_Good-BsmBI-F	CGTCTCGAGATCTATTGCACAAGTTTTAAAACCA	For deleting gene *pyrE* in *C. butyricum* by AsCas12a or FnCas12a, amplifying the fragment of the target for Good_Pre-crRNA recognizing 5’-GTTA PAM
54	c.bDpyrE-As_GTTA_Good-BsmBI-R	CGTCTCGATTATGGTTTTAAAACTTGTGCAATAG	
55	c.bDpyrE-Fn_GTTA_Good-BsmBI-R	CGTCTCGAATTTGGTTTTAAAACTTGTGCAATAG	
56	c.bDpyrE-GTTA_Bad-BsmBI-F	CGTCTCGAGATCAACAGCAGGAACTTCG	For deleting gene *pyrE* in *C. butyricum* by FnCas12a, amplifying the fragment of the target for Bad_Pre-crRNA recognizing 5’-GTTA PAM
	c.bDpyrE-Fn_GTTA_Bad-BsmBI-R	CGTCTCGAATTATAAATCGAAGTTCCTGCTGTTGATCT	
57	c.bDspo0A-CTTA_Good-BsmBI-F	CGTCTCGAGATTCCAACAACTTCTTCATATATTC	For deleting gene *spo0A* in *C. butyricum* by AsCas12a or FnCas12a, amplifying the fragment of the target for Good_Pre-crRNA recognizing 5’-CTTA PAM
58	c.bDspo0A-As_CTTA_Good-BsmBI-R	CGTCTCGATTAGAATATATGAAGAAGTTGTTGGAATCTC	
59	c.bDspo0A-Fn_CTTA_Good-BsmBI-R	CGTCTCGAATTGAATATATGAAGAAGTTGTTGGAATCTC	
60	c.bDspo0A-CTTA_Bad-BsmBI-F	CGTCTCGAGATTTCTTTTTGTAAATACTTCCATGAATT	For deleting gene *spo0A* in *C. butyricum* by FnCas12a, amplifying the fragment of the target for Bad_Pre-crRNA recognizing 5’-CTTA PAM
61	c.bDspo0A-Fn_CTTA_Bad-BsmBI-R	CGTCTCGAATTCATGGAAGTATTTACAAAAAGAAATCTCG	
62	c.bDspo0A-ATTA_Good-BsmBI-F	CGTCTCGAGATCAAACATAATTCATGAAATCGGT	For deleting gene *spo0A* in *C. butyricum* by AsCas12a or FnCas12a, amplifying the fragment of the target for Good_Pre-crRNA recognizing 5’-ATTA PAM
63	c.bDspo0A-As_ATTA_Good-BsmBI-R	CGTCTCGATTAACCGATTTCATGAATTATGTTTG	
64	c.bDspo0A-Fn_ATTA_Good-BsmBI-R	CGTCTCGAATTACCGATTTCATGAATTATGTTTG	
65	c.bDspo0A-ATTA_Bad-BsmBI-F	CGTCTCGAGATATTGCTTCTATTTGTCCTCTGC	For deleting gene *spo0A* in *C. butyricum* by FnCas12a, amplifying the fragment of the target for Bad_Pre-crRNA recognizing 5’-ATTA PAM
66	c.bDspo0A-Fn_ATTA_Bad-BsmBI-R	CGTCTCGAATTGGCAGAGGACAAATAGAAGC	

67	c.bDspo0A-GTTA_Good-BsmBI-F	CGTCTCGAGATTTCTTGATATCATAATGCCACAT	For deleting gene *spo0A* in *C. butyricum* by AsCas12a or FnCas12a, amplifying the fragments of the target for Good_Pre-crRNA recognizing 5’-GTTA PAM
68	c.bDspo0A-As_GTTA_Good-BsmBI-R	CGTCTCGATTAATGTGGCATTATGATATCAAGAA	
69	c.bDspo0A-Fn_GTTA_Good-BsmBI-R	CGTCTCGAATTATGTGGCATTATGATATCAAGAA	
70	c.bDspo0A-GTTA_Bad-BsmBI-F	CGTCTCGAGATCGGGGATTGCAAAAGATG	For deleting gene *spo0A* in *C. butyricum* by FnCas12a, amplifying the fragment of the target for Bad_Pre-crRNA recognizing 5’-GTTA PAM
71	c.bDspo0A-Fn_GTTA_Bad-BsmBI-R	CGTCTCGAATTTCTACCATCTTTTGCAATCCCCGATCT	
72	DuidA-U-AatII-F	ATTGGCGACGTCACCGAGG	Amplifying the fragment upstream for deleting gene *uidA* from the genome of *E. coli* MG1655 RARE
73	DuidA-U-BsaI-R	GGTCTCTGAAAGAACTGTACAGCGAA	
74	DuidA-D-BsaI-F	GGTCTCATTTCATTAAGATAATAATACTGGTCAACCT	Amplifying the fragment downstream for deleting gene *uidA* from the genome of *E. coli* MG1655 RARE
75	DuidA-D-SalI-R	AGGCGTCGACTTCCGGGAACCGATTG	
76	DuidA-F	TCACACTGTCCACCACTCGTCC	Colony PCR for deleting gene *uidA* in *E. coli* MG1655 RARE
77	DuidA-R	GCTCACCTGACAACGCGGTAAA	
78	DuidA-Fn_ATTA_Good-BsmBI-F	CGTCTCGAGATACCACAAACCGTTCTACTTTACT	For deleting gene *uidA* in *E. coli* MG1655 RARE by FnCas12a, amplifying the fragment of the target for Good_Pre-crRNA recognizing 5’-ATTA PAM
79	DuidA-Fn_ATTA_Good-BsmBI-R	CGTCTCGAATTAGTAAAGTAGAACGGTTTGTGGTATCT	
80	DuidA-Fn_ATTA_Bad-BsmBI-F	CGTCTCGAGATCGCTGCGATGGATC	For deleting gene *uidA* in *E. coli* MG1655 RARE by FnCas12a, amplifying the fragment of the target for Bad_Pre-crRNA recognizing 5’-ATTA PAM
81	DuidA-Fn_ATTA_Bad-BsmBI-R	CGTCTCGAATTCTATGCCGGGATCCATCGCAGCGATCT	
82	c.bDspo0A-TTTG_Good-BsmBI-F	CGTCTCGAGATCAATCCCCGTAACCAC	For deleting gene *spo0A* in *C. butyricum* by AsCas12a or FnCas12a, amplifying the fragments of the target for Good_Pre-crRNA recognizing 5’-TTTG PAM
83	c.bDspo0A-As_TTTG_Good-BsmBI-R	CGTCTCGATTAAGATATTGTGGTTACGGGGATTGATCT	
84	c.bDspo0A-Fn_TTTG_Good-BsmBI-R	CGTCTCGAATTAGATATTGTGGTTACGGGGATTGATCT	
85	c.bDspo0A-TTTC_Good-BsmBI-F	CGTCTCGAGATCAACACCCCTAAACCATC	For deleting gene *spo0A* in *C. butyricum* by AsCas12a or FnCas12a, amplifying the fragments of the target for Good_Pre-crRNA recognizing 5’-TTTC PAM
86	c.bDspo0A-As_TTTC_Good-BsmBI-R	CGTCTCGATTAATCTTGATGGTTTAGGGGTGTTGATCT	
87	c.bDspo0A-Fn_TTTC_Good-BsmBI-R	CGTCTCGAATTATCTTGATGGTTTAGGGGTGTTGATCT	
88	c.bDspo0A-TTTT_Good-BsmBI-F	CGTCTCGAGATCCAACACCCCTAAACC	For deleting gene *spo0A* in *C. butyricum* by AsCas12a or

89	c.bDspo0A-As_TTTT_Good-BsmBI-R	CGTCTCGATTATCTTGATGGTTTAGGGGTGTTGGATCT	FnCas12a, amplifying the fragments of the target for Good_Pre-crRNA recognizing 5’-TTTT PAM
90	c.bDspo0A-Fn_TTTT_Good-BsmBI-R	CGTCTCGAATTTCTTGATGGTTTAGGGGTGTTGGATCT	
91	c.bDpyrE-TTTG_Good-BsmBI-F	CGTCTCGAGATTAACAAAATCTCCGAATGTCAAC	For deleting gene *pyrE* in *C. butyricum* by AsCas12a or FnCas12a, amplifying the fragments of the target for Good_Pre-crRNA recognizing 5’-TTTG PAM
92	c.bDpyrE-As_TTTG_Good-BsmBI-R	CGTCTCGATTAGTTGACATTCGGAGATTTTGTTAATCTC	
93	c.bDpyrE-Fn_TTTG_Good-BsmBI-R	CGTCTCGAATTGTTGACATTCGGAGATTTTGTTA	
94	c.bDpyrE-TTTC_Good-BsmBI-F	CGTCTCGAGATATCTTTTATTCCTCCGTTAACTT	For deleting gene *pyrE* in *C. butyricum* by AsCas12a or FnCas12a, amplifying the fragments of the target for Good_Pre-crRNA recognizing 5’-TTTC PAM
95	c.bDpyrE-As_TTTC_Good-BsmBI-R	CGTCTCGATTAAAGTTAACGGAGGAATAAAAGATATCT	
96	c.bDpyrE-Fn_TTTC_Good-BsmBI-R	CGTCTCGAATTGTTGACATTCGGAGATTTTGTTA	
97	c.bDpyrE-As_TTTT_Good-BsmBI-F	CGTCTCGAGATACATCAACATCTCCTTGGC	For deleting gene *pyrE* in *C. butyricum* by AsCas12a, amplifying the fragment of the target for Good_Pre-crRNA recognizing 5’-TTTT PAM
98	c.bDpyrE-As_TTTT_Good-BsmBI-R	CGTCTCGATTAAAGAGCCAAGGAGATGTTGATGTATCT	
99	c.bDpyrE-Fn_TTTT_Good-BsmBI-F	CGTCTCGAGATCTATTAGAACAATACTTAACATCAATT	For deleting gene *pyrE* in *C. butyricum* by FnCas12a, amplifying the fragment of the target for Good_Pre-crRNA recognizing 5’-TTTT PAM
100	c.bDpyrE-Fn_TTTT_Good-BsmBI-R	CGTCTCGAATTGATGTTAAGTATTGTTCTAATAGATC	
101	uni_IN-F-BsaI	GGTCTCGTGGAGGATAATCAATCGTCCCTTC	Amplifying the fragments of the gene expression cassette from the derivative vectors of pGG2121
102	uni_IN-R-BsaI	GGTCTCATCAGAACGGCGCGCC	
103	Inc.bGusA_1.5k-U-AatII-F	TGTGTAGACGTCATATGTAAAACAGAAAGAAGTGATAG	For integrating the GusA/IFP2.0 expression cassette in *C. butyricum*, amplifying the fragment upstream from the genome of *C. butyricum* DSM10702
104	Inc.bGusA_1.5k-U-BsaI-R	GGTCTCGTCCACAATTTGTTCAATTTTAGAATTGCG	
105	Inc.bGusA_1.5k-D-BsaI-F	GGTCTCTCTGATGTATAAATTGCATAAGCTTACCG	For integrating the GusA/IFP2.0 expression cassette in *C. butyricum*, amplifying the fragment downstream from the genome of *C. butyricum* DSM10702
106	Inc.bGusA_1.5k-D-SalI-R	AGGCGTCGACAATTGGGATACTTTGAAACATACAG	
107	Inc.bGusA-F	tggaccagtaccaaatggagga	Colony PCR for integrating the GusA/IFP2.0 expression cassette in *C. butyricum*
108	Inc.bGusA-R	gaggttactgactacgtggttg	
109	Inc.bGusA-T1-BsmBI-F	CGTCTCGAGATTAATCACTCCACACATTACATTAAATT	For integrating the GusA expression cassette in *C. butyricum*, amplifying the fragment of the target T1
110	Inc.bGusA-T1-BsmBI-R	CGTCTCGAATTTAATGTAATGTGTGGAGTGATTA	
111	Inc.bGusA-T2-BsmBI-F	CGTCTCGAGATCAATTTCATCCGTTTCATCTAAGAAT	For integrating the GusA/IFP2.0 expression cassette in *C. butyricum*,

112	Inc.bGusA-T2-BsmBI-R	CGTCTCGAATTCTTAGATGAAACGGATGAAATTGATCT	amplifying the fragment of the target T2
113	Inc.bGusA_1.0k-AatII-F	GCATCCGACGTCCCTAAGATACCAGAGGATCC	For integrating the GusA expression cassette, amplifying the fragment of the donor DNA template of 1.0k donor arms from vector pP*fet*Fn-Inc.bGusA_1.5k-Tn
114	Inc.bGusA_1.0k-SalI-R	AGGCGTCGACGATAGTAGACATTGTATCTATGTATTTG	
115	Inc.bGusA_0.75k-AatII-F	TACTAGGACGTCGAATATATACTGAGTGGTATAGAGGT	For integrating the GusA expression cassette, amplifying the fragment of the donor DNA template of 0.75k donor arms from vector pP*fet*Fn-Inc.bGusA_1.5k-Tn
116	Inc.bGusA_0.75k-SalI-R	AGGCGTCGACATTGTAGATAAAACAGGTAAAGAG	
117	Inc.bGusA_0.5k-AatII-F	AGAATTGACGTCGTAAGGTACCATATGAGATTTTGA	For integrating the GusA expression cassette, amplifying the fragment of the donor DNA template of 0.5k donor arms from vector pP*fet*Fn-Inc.bGusA_1.5k-Tn
118	Inc.bGusA_0.5k-SalI-R	AGGCGTCGACACTAATTGAAATAAAAAGAGCTATAAAAG	
119	InspIFP-U-AatII-F	TGTGTAGACGTCGCTATCAGATGATGATGATGATAT	For integrating the IFP2.0 expression cassette in *C. sporogenes*, amplifying the fragment upstream from the genome of *C. sporogenes* NCIMB 10696
120	InspIFP-U-BsaI-R	GGTCTCGTCCACAAATTTAGAACTTCTTAGCTGTAT	
121	InspIFP-D-BsaI-F	GGTCTCTCTGAGAAAGTTATTTAAATCTTAGAACTCTTAGT	For integrating the IFP2.0 expression cassette in *C. sporogenes*, amplifying the fragment downstream from the genome of *C. sporogenes* NCIMB 10696
122	InspIFP-D-SalI-R	AGGCGTCGACAAACTGAACTTAATAACGTGGT	
123	InspIFP-F	AAATCGGGGAGTCTCTTTGACA	Colony PCR for integrating the IFP2.0 expression cassette in *C. sporogenes*
124	InspIFP-R	CTTCCCTATGAAGAAAACTATTTCC	
125	InspIFP-T1-BsmBI-F	CGTCTCGAGATTAATCACTCCACACATTACATTAAATT	For integrating the IFP2.0 expression cassette in *C. sporogenes*, amplifying the fragment of the target T1
126	InspIFP-T1-BsmBI-R	CGTCTCGAATTTAATGTAATGTGTGGAGTGATTA	
127	Inc.bHemO-U-AatII-F	TGTGTAGACGTCGCATCTAAAAATTTTACAGAGCA	For integrating the HemO expression cassette with promoter P*thl*_fU/P*thl*79%-26_fU in *C. butyricum*, amplifying the fragment upstream from the genome of *C. butyricum* DSM10702
128	Inc.bHemO-U-BsaI-R	GGTCTCGTCCAACGTTAATTTATATATCCTTCTGATACT	
129	Inc.bHemO-D-BsaI-F	GGTCTCTCTGACAATCCAAAAGTTGTGTATAAATACT	For integrating the HemO expression cassette with promoter P*thl*_fU/P*thl*79%-26_fU in *C. butyricum*, amplifying the fragment downstream from the genome of *C. butyricum* DSM10702
130	Inc.bHemO-D-SalI-R	AGGCGTCGACTGTTGGTATTGCAGAAGAAC	
131	Inc.bHemO-F	ATTGCAGCCTTTGTTGGAGCTGG	Colony PCR for integrating the HemO expression cassette with

132	Inc.bHemO-R	AGGAAAAGGATTTGAACTTGCTGA	promoter P*thl*_fU/P*thl*79%-26_fU in *C. butyricum*
133	Inc.bHemO-T1-BsmBI-F	CGTCTCGAGATGGAAAGAAAGAATCTAAGAAGAAAATT	For integrating the HemO expression cassette with promoter P*thl*_fU/P*thl*79%-26_fU in *C. butyricum*, amplifying the fragment of the target T1
134	Inc.bHemO-T1-BsmBI-R	CGTCTCGAATTTTCTTCTTAGATTCTTTCTTTCCATCT	
135	InspHemO-U-AatII-F	TGTGTAGACGTCGAAATGGAAGATATAGCGGGA	For integrating the HemO expression cassette with promoter P*thl*_fU/P*thl*79%-26_fU in *C. sporogenes*, amplifying the fragment of the upstream from genome of *C. sporogenes* NCIMB 10696
136	InspHemO-U-BsaI-R	GGTCTCGTCCACTATCTTCACTACCGGCTC	
137	InspHemO-D-BsaI-F	GGTCTCTCTGATAAATTTTATTTAGCAACTATATTAACTAATCT	For integrating the HemO expression cassette with promoter P*thl*_fU/P*thl*79%-26_fU in *C. sporogenes*, amplifying the fragment downstream from the genome of *C. sporogenes* NCIMB 10696
138	InspHemO-D-SalI-R	AGGCGTCGACATATGGGATATCTAAACTTTGATGAA	
139	InspHemO-F	CTTTTAACCATTCAGCACTATTTGCT	Colony PCR for integrating the HemO expression cassette with promoter P*thl*_fU/P*thl*79%-26_fU in *C. sporogenes*
140	InspHemO-R	ACAGTGACGCTGCTTTTGATCCT	
141	InspHemO-T1-BsmBI-F	CGTCTCGAGATAAAGACTGTCTCAAAATAAAAATAATT	For integrating the HemO expression cassette with promoter P*thl*_fU/P*thl*79%-26_fU in *C. sporogenes*, amplifying the fragment of the target T1
142	InspHemO-T1-BsmBI-R	CGTCTCGAATTATTTTTATTTTGAGACAGTCTTTATCT	
143	hemL-BsaI-F	GGTCTCCACATATGACTAAAAATGATCAAATTTTTACT	Amplifying fragment of the *hemL* gene from the genome of *C. butyricum* DSM10702
144	hemL-BsaI-R	GGTCTCACCTTTTAATTTGATTCACTAACTGCAAAT	
145	RBS-hemA-BsaI-F	GGTCTCCAAGGAGGTGTGTTACATATGATAGGGTTAATAGGAATTAAAAGA	Amplifying fragment of the *hemA* gene with RBS sequence from the genome of *C. butyricum* DSM10702
146	hemA-BsaI-R	GGTCTCAGTCTCTACTTCATCCTGAATATCTTCTC	
147	InspHemLA-U-AatII-F	TGTGTAGACGTCCATTTGATAAATTTGACCTAAAACAA	For integrating the HemLA polycistronic expression cassette in *C. sporogenes*, amplifying the fragment upstream from the genome of *C. sporogenes* NCIMB 10696
148	InspHemLA-U-BsaI-R	GGTCTCGTCCACAAATATTATGTCTCACAAAATTTTATTG	
149	InspHemLA-D-BsaI-F	GGTCTCTCTGATGAGCAGGTTCACTTTTGT	For integrating the HemLA polycistronic expression cassette in *C. sporogenes*, amplifying the fragment downstream from the genome of *C. sporogenes* NCIMB 10696
150	InspHemLA-D-SalI-R	AGGAGTCGACGGAGCAAGTGAATATCCATTT	
151	InspHemLA-F	TCGTGGTATGGGATATAGGTTCA	Colony PCR for integrating the HemLA polycistronic expression cassette in *C. sporogenes*
152	InspHemLA-R	AAGAGTTCGCTGTGGTGGTCAT	

153	InspHemLA-T1-BsmBI-F	CGTCTCGAGATAACTTAATTTTTATATCAATCTAAATT	For integrating the HemLA polycistronic expression cassette in *C. sporogenes*, amplifying the fragment of the target T1
154	InspHemLA-T1-BsmBI-R	CGTCTCGAATTTAGATTGATATAAAAATTAAGTTATCT	
155	uni_IN-F-BsmBI	CGTCTCATGGAGGATAATCAATCGTCCCTTC	Alternative to the primer pair uni_IN-F/R-BsaI, amplifying the fragments of the gene expression cassette from the derivative vectors of pGG2121
156	uni_IN-R-BsmBI	CGTCTCATCAGCCATTAGCTAAGGATTCAGAA	
157	InspHemNG-U-AatII-F	TGTGTAGACGTCTAGTTGGAGTAATTGGTATGGA	For integrating the HemNG polycistronic expression cassette in *C. sporogenes*, amplifying the fragment upstream from the genome of *C. sporogenes* NCIMB 10696
158	InspHemNG-U-BsmBI-R	CGTCTCGTCCAGGATAACGACTTCTAAGCTTTA	
159	InspHemNG-D-BsmBI-F	CGTCTCTCTGACCAAATTAAGAACTACTAAAGCTT	For integrating the HemNG polycistronic expression cassette in *C. sporogenes*, amplifying the fragment downstream from the genome of *C. sporogenes* NCIMB 10696
160	InspHemNG-D-SalI-R	AGGAGTCGACCAGAATCAAGAGTATATTAGGTGC	
161	InspHemNG-F	GTAGAAATGAAAGGAGATCCTA	Colony PCR for integrating the HemNG polycistronic expression cassette in *C. sporogenes*
162	InspHemNG-R	GGGATACTTATACCTTTTTACG	
163	InspHemNG-T1-BsmBI-F	CGTCTCGAGATTCTCAAAGTAAAAATAGATTTAAAATT	For integrating the HemNG polycistronic expression cassette in *C. sporogenes*, amplifying the fragment of the target T1
164	InspHemNG-T1-BsmBI-R	CGTCTCGAATTTTAAATCTATTTTTACTTTGAGAATCT	
165	InspHemEH-U-AatII-F	TGTGTAGACGTCCTTATGAGTACAAACAATTATAATGTTAT	For integrating the HemEH polycistronic expression cassette in *C. sporogenes*, amplifying the fragment upstream from the genome of *C. sporogenes* NCIMB 10696
166	InspHemEH-U-BsmBI-R	CGTCTCGTCCACACCACCAATATAAAAAGTATATTCAT	
167	InspHemEH-D-BsmBI-F	CGTCTCTCTGATTAAATTTTAAATATATTTATATCTTTTTCAAGTTTTA	For integrating the HemEH polycistronic expression cassette in *C. sporogenes*, amplifying the fragment downstream from the genome of *C. sporogenes* NCIMB 10696
168	InspHemEH-D-SalI-R	AGGAGTCGACAGTAATATAACTATGGTATCAACTTCT	
169	InspHemEH-F	GACCTATAATCAGGATTTTTAGAGATAATGA	Colony PCR for integrating the HemEH polycistronic expression cassette in *C. sporogenes*
170	InspHemEH-R	GTAGTTTACACCGATAAGTATAGCAC	
171	InspHemEH-T1-BsmBI-F	CGTCTCGAGATTATTCTCTTATTTTGATACAATTAATT	For integrating the HemEH polycistronic expression cassette in *C. sporogenes*, amplifying the fragment of the target T1
172	InspHemEH-T1-BsmBI-R	CGTCTCGAATTAATTGTATCAAAATAAGAGAATAATCT	

Table S3. Sequences of gBlock fragments codon-optimized and synthesized by Integrated DNA Technologies. The open reading frames are highlighted in bold. Restriction sites are underlined.

gBlock fragments	5' - 3' Sequences
COtetR-PIPL12<br>(Promoter miniP*4*_tU;<br>P*IPL12*; Tet operator<br>sequence)	GGTCTCGTCCA**TTAAGACCCACTTTCACATTTAAGTTGTTTTTCTAAT**<br>**CCGCAAATTATTAATTCAAGACCGAATAAAAATGCTGGCTCTGCAC**<br>**CTTGGTGATCAAATAATTCTATAGCTTGCCTTAATAATGGTGGCATA**<br>**CTATCAGTAGTAGGTGTTTCCCTTTCTTCTTTAGCAACTTGATGCTC**<br>**TTGATCTTCCAATACGCAACCTAAAGTAAAATGCCCCACAGCGCTT**<br>**AATGCATATAATGCATTCTCTAGTGAAAAACCTTGTTGGCATAAAAA**<br>**GGCTAATTGATTTTCTAAAGTTTCATACTGTTTTTCTGTAGGTCTTG**<br>**TACCTAAATGTACTTTTGCTCCATCCCTATGACTAAGTAAAGCACAT**<br>**CTAAAACTTTTAGCGTTATTTCTTAAAAAATCTTGCCAGCTTTCCCC**<br>**TTCTAAAGGGCAAAAGTGAGTATGATGTCTATCTAACATCTCAATA**<br>**GCTAAGGCATCCAATAAAGCTCTCTTATTTTTTACATGCCAATACAA**<br>**TGTAGGCTGCTCTACACCCAACTTCTGAGCTAATTTTCTGGTTGTTA**<br>**AACCTTCTATTCCTACCTCATTAAGTAACTCTAATGCTGAGTTAATT**<br>**ACTTTACTTTTATCTAATCTTGACAT**TCTAACTAACCTCCTAAATTTTG<br>ATACGGGGTAACAGATAAACCATTTCAATCTATTTCATAAGTTCCATAG<br>TTTATCCCTAATTTATACGTTTCTCTAACAACTTAATTTTAACTTTAAA<br>AAATAAATTTGTCAAAAATTATAAGTATTGAGCTTCTATCATTGATAGG<br>TTATAATGAACATCGTATAAAGTTGTGTAATTTTTAAGGAGGTGTGTTA<br>CATATGTCGAGACC
COtetR-Pfet<br>(Promoter miniP*4*_tU;<br>P*fet*; Tet operator<br>sequence)	GGTCTCGTCCA**TTAAGACCCACTTTCACATTTAAGTTGTTTTTCTAAT**<br>**CCGCAAATTATTAATTCAAGACCGAATAAAAATGCTGGCTCTGCAC**<br>**CTTGGTGATCAAATAATTCTATAGCTTGCCTTAATAATGGTGGCATA**<br>**CTATCAGTAGTAGGTGTTTCCCTTTCTTCTTTAGCAACTTGATGCTC**<br>**TTGATCTTCCAATACGCAACCTAAAGTAAAATGCCCCACAGCGCTT**<br>**AATGCATATAATGCATTCTCTAGTGAAAAACCTTGTTGGCATAAAAA**<br>**GGCTAATTGATTTTCTAAAGTTTCATACTGTTTTTCTGTAGGTCTTG**<br>**TACCTAAATGTACTTTTGCTCCATCCCTATGACTAAGTAAAGCACAT**<br>**CTAAAACTTTTAGCGTTATTTCTTAAAAAATCTTGCCAGCTTTCCCC**<br>**TTCTAAAGGGCAAAAGTGAGTATGATGTCTATCTAACATCTCAATA**<br>**GCTAAGGCATCCAATAAAGCTCTCTTATTTTTTACATGCCAATACAA**<br>**TGTAGGCTGCTCTACACCCAACTTCTGAGCTAATTTTCTGGTTGTTA**<br>**AACCTTCTATTCCTACCTCATTAAGTAACTCTAATGCTGAGTTAATT**<br>**ACTTTACTTTTATCTAATCTTGACAT**TCTAACTAACCTCCTAAATTTTG<br>ATACGGGGTAACAGATAAACCATTTCAATCTATTTCATAAGTTCCATAG<br>TTTATCCCTAATTTATACGTTTCTCTAACAACTTAATTTTAACTTTAAA<br>AAATAAATTTGTCAAAAATTACTTTAAAATCTATCATTGATAGGGTAAA<br>ATATAAATCGTATAAAGTTGTGTAATTTTTAAGGAGGTGTGTTACATAT<br>GTCGAGACC
IFP2.0-BsaI	GGTCTCCACAT**ATGGCAAGAGATCCTCAACCTTTTTTTCCTCCACTTT**<br>**ATTTAGGAGGACCAGAAATAACAACTGAAAATTGTGAAAGAGAACC**<br>**TATTCATATTCCAGGATCAATTCAACCTCATGGTGCTTTATTAACTG**<br>**CTGATGGACATTCAGGAGAAGTACTTCAAGTAAGTCTTAATGCTGC**<br>**AACTTTTCTTGGACAAGAACCAACTGTACTTAGAGGACAAACATTA**<br>**GCAGCATTATTACCTGATCAATGGCCAGCTTTACAAACAGCTCTTCC**<br>**ACCTGGTTGTCAAGATGCTCTTCAATATAGAGCTACATTAGATTGG**<br>**CCTGCTGCTGGTCATTTATCACTTACTGTACATAGAGTTGCTGAATT**<br>**ATTAATATTAGAATTTGAACCTACTGAAGCTTGGGATTCAATTGGAC**<br>**CACATGCACTTAGAAATGCTATGTTTGCTTTAGAATCAGCTCCAAAT**<br>**CTTAGAGCATTAGCTGAAGTTGCAACTCAAACAGTTAGAGAACTTT**<br>**CTGGATTTGATAGAGTAATGTTATATAAATTTGCACCTGATGCTACA**<br>**GGTGAAGTTATAGCAGAAGCTAGAAGAGAAGGAATGCAAGCTTATC**<br>**TAGGTCATAGATTTCCAGCTAGTACAACTCCTGCTCAAGCAAGAGC**

	**TTTATATACAAGACATCTTTTAAGATTAACAGCAGATACTAGAGCAG**<br>**CTGCTGTTCCTTTAGATCCAGTTTTAAATCCACAAACAAATGCTCCT**<br>**ACACCTCTTGGTGGTGCTGTACTAAGAGCAACTTCACCTATGCATA**<br>**TGCAATATCTAAGAAATATGGGAGTTGGTTCAAGTTTATCAGTTTCT**<br>**GTAGTTGTTGGAGGTCAATTATGGGGTCTTATTGTATGTCATCATCA**<br>**AACACCTTATGTTTTACCTCCTGATTTAAGAACTACTCTTGAATATC**<br>**TTGGTAGATTATTATCTCTACAAGTTCAAAGAAAAGAAGCATAA**AGA<br>CAGAGACC
UnaG-BsaI	GGTCTCCACAT**ATGGTTGAAAAGTTTGTAGGAACATGGAAGATTGCA**<br>**GATTCACATAATTTTGGTGAATATTTAAAAGCTATAGGTGCTCCTAA**<br>**AGAATTAAGTGATGGTGGAGATGCTACTACTCCTACATTATATATTT**<br>**CACAAAAGGATGGTGATAAGATGACTGTTAAGATTGAAAATGGACC**<br>**TCCTACTTTTCTTGATACTCAAGTTAAGTTTAAGCTTGGAGAAGAAT**<br>**TTGATGAATTTCCTTCAGATAGAAGAAAAGGTGTAAAATCAGTAGT**<br>**TAATTTAGTAGGAGAAAAATTAGTTTATGTACAAAAGTGGGATGGT**<br>**AAAGAAACTACTTATGTTAGAGAAATTAAAGATGGAAAGTTAGTTG**<br>**TAACTTTAACTATGGGTGATGTTGTTGCTGTTAGATCATATAGAAGA**<br>**GCTACAGAATAA**AGACAGAGACC
CreiLOV-BsaI	GGTCTCCACAT**ATGGCAGGATTAAGACATACATTTGTTGTTGCTGAT**<br>**GCAACACTTCCAGATTGTCCTCTTGTTTATGCATCAGAAGGTTTTTA**<br>**TGCTATGACTGGATATGGACCTGATGAAGTTTTAGGTCATAATGCT**<br>**AGATTTCTTCAAGGAGAAGGTACAGATCCAAAAGAAGTACAAAAAA**<br>**TTAGAGATGCTATTAAAAAAGGTGAAGCTTGTTCAGTAAGATTATTA**<br>**AATTATAGAAAAGATGGTACTCCTTTTTGGAATCTTTTAACTGTTAC**<br>**ACCAATTAAAACTCCTGATGGAAGAGTATCAAAATTTGTAGGTGTT**<br>**CAAGTTGATGTTACTTCTAAAACTGAAGGAAAGGCATTAGCTTAA**A<br>GACAGAGACC
HemO-BsaI	CGGGTCTCCACAT**ATGATAAATGAATTTATGAAGAAAATAAGATTTG**<br>**AAAGTGAATCATTACATGATATGGCTGAACATACAGGTTTTATTAAT**<br>**AGATTAATTGAAGGAAATGCTAGTAAGGAAACTTATGGAAAGTATA**<br>**TTTACAATCTTTATCATGTATATAAAGCTATTGAAGATAATCTTGAA**<br>**AAGAATAAATCAAATGAAAATGTAGCTAATTTTGCATTACCTGATGT**<br>**TTATAGATCAGAAGAAATTTCAAAAGATGTTAAGTCAATACTTGGTG**<br>**AGGATTATGAAAAAGTTCCTTTACTTATGAGTACAAAGGTATTTGTA**<br>**AATAGAATTAATTTTATAGGTAATAGTGATCCTGAACTTTTAATAGC**<br>**TCATGCTTATACTAGATATTTAGCAGATTTATTTGGTGGTAGAACTA**<br>**TACTTGAAATAATAAAAAAACATTATAAGTTAGAAGATGAAAGTCTT**<br>**AATTATTATGTATTTCCACAAATTAAAGATTTTAGACAATTTGTTAT**<br>**GCAATATCATGGTAAGTTAAATGCACTTAATTTATCAGAATCAATGC**<br>**AAGAAAAATTTCTTAATGAAATTAGTATATCATATATATACAATATA**<br>**AGTATTTCTAATGAACTTGAATTTTTAGAATACCATAAGAAATAA**AG<br>ACGGAGACC
HemN-HemG-BsmBI	CGTCTCCTCCA**TTATTTAAGAGTAGGCTTATCTGTTAAATGAGCTATT**<br>**TCTCTTGCAAAATTAGCTACTTGTTCCCAATCAGTATAAACAACTTC**<br>**CTTTCTAGTATCAGTTTCTCCACCAGACATCTTCATTATTAACTTAA**<br>**TCATAAATCTATCATACCATCTATATCTTGGATATCTAAGAGCACCA**<br>**GCAATTACAGCACATCTATCTGGTCTCCATTGACTATTCATTAAAAA**<br>**CTTTCTTGCATAACTATTTGTTTGAGGTGTTCTCTTTTCAGGTTTTC**<br>**TAGCAACTAAATTAACACTATAAAAAGCACTTGGCATACTATTAAGT**<br>**CTAGTAGCATGCTTTTTTACAAATTCTTGAAAAGCTGAATGATAATG**<br>**ACCATATCTTATACTAGCTCCTATAACAACTCTATCATAATTTTCCC**<br>**ATTGTGGTTCTTCTATTCTATGTACATTTGCTACATCTGCTTGAATA**<br>**CCAAGTTCTTTTAATTCACTTGCTAAATAACTAGCAATTTCTCTAGT**<br>**TTGACCATCTCTTGTAGAAAATAAAATTAATGTCTTCAT**ATGTAACA<br>CACCTCCTT**TTATATAACTCTAGAAAATTGTTGCATTCTTGCCTTTTG**<br>**TCTTAAATAAGTATCAAAACACATACAAATATTTCTTATAAGAAGTC**<br>**TTCCTTTAGCAGTAACTTGTATTCCCTTTTCATCAACATCTACAAGT**<br>**CCATCTTTAGCTAAAGGAGCTAAAAGTTTAAGATCTTCAGCAAAATA**<br>**ATCTGCAAAATGTAAATCCCATTGTTTTTCAATAGGTGCATAATCAA**<br>**GTCTAAAATTACATATTAATGATTTTATAACATCTCTTCTAATACAA**

	**TCATCTCTAGTTAATGCAATTCCTCTCCAAAGTGCATTTCCTTGTTC**<br>**ATCTACTTGTTGATAATATTGCTTAAGTTCTTTTTGATTTTGTGCAT**<br>**AACAATCTCCAATCATTGAAATTGCTGATACACCCATACCAAGAAG**<br>**ATCTGTATCACCTTGTGTTGTATAACCTTGAAAATTTCTATGTAAAA**<br>**CTCCTTCTCTTTGTGCTACTGCAAGTTCATCATCAGGTCTAGCAAAA**<br>**TGATCCATTCCTATAAATTGATAACCAGATTGTGTAAGAAATGCTAT**<br>**AGTTTCTTGAAGAATATCAAGTTTTTGTTGAGGTGAAGGTAAATCA**<br>**GCATCTTTTATTTTTCTTTGAGCAGCAAATATAGTAGGAAGATGAGC**<br>**ATAATTAAAAACAGAAAGTCTATCTGGATTTAATTCTGCTACTCTTT**<br>**TAAGAGTAAATGCAAAACTTTCAGGTGTTTGCTTAGGTAAACCATAT**<br>**ATAAGATCAATATTAGTTGAAGTAAAACCAATTTCTCTAGCATGATT**<br>**AAGAAGAGCAAAAATAAATTCTTCATCTTGTTCTCTATTTACTAATC**<br>**TTTGTACTTCCTTATTAAAATCTTGTACTCCCATACTTAATCTATTAA**<br>**ATCCTTCTGCTCTAAGATGATCAAGTACATCTAATTCTATTTCTCTT**<br>**GGATCAACTTCTATACTAATTTCTGCATCAGCATTAAATTGAAAATT**<br>**TTCTCTTAATAATTTCATAAGTCTACTTATTTGAGCCTTATTTAAATA**<br>**TGTAGGAGTACCTCCTCCCCAATGTAATTGTGAAACATGTCTACCT**<br>**GCAAATAATGGAGCTCTATGAACAATTTCTTGTTCCAATGCATCAAG**<br>**ATATTGATCTGCTTTATGTTGTTGTCTTGTTACAATCTTATTACAAC**<br>**CACAAAAATAACATAACTTATGACAAAAAGGTATATGTACATATAAA**<br>**GATAAAGGTCTTTCTGGATATCTAGCTACAGCTTGTAAAAATGCTTG**<br>**TTCACCAAAATCTTCACTAAATTCAAGAGCTGTTGGATAACTAGTAT**<br>**ATCTTGGTCCACTATAATTATACTTTTGAATTAAAGCAAGATCCCAA**<br>**TCAATTTGTTGTACTGACAT**ATGTTGAGACG
HemE-HemH-BsmBI	CGTCTCCACAT**ATGACTGAATTAAAGAATGATAGATATTTAAGAGCT**<br>**CTTCTTAGACAACCAGTTGATGTTACTCCAGTTTGGATGATGAGAC**<br>**AAGCTGGTAGATATCTTCCTGAATATAAGGCAACTAGAGCACAAGC**<br>**AGGAGATTTTATGTCTCTTTGTAAAAATGCAGAATTAGCATGTGAA**<br>**GTTACATTACAACCTCTTAGAAGATATCCATTAGATGCAGCAATTCT**<br>**TTTTAGTGATATATTAACTGTACCTGATGCAATGGGTTTAGGTTTAT**<br>**ATTTTGAAGCAGGTGAAGGACCTAGATTTACATCTCCTGTTACTTGT**<br>**AAAGCTGATGTTGATAAGTTACCTATACCAGATCCTGAAGATGAAT**<br>**TAGGTTATGTAATGAATGCTGTTAGAACTATAAGAAGAGAACTTAA**<br>**AGGAGAAGTACCTTTAATAGGTTTTTCAGGATCACCTTGGACTTTA**<br>**GCAACTTATATGGTTGAAGGAGGTTCTTCTAAGGCATTTACTGTTAT**<br>**AAAAAAGATGATGTATGCAGATCCACAAGCTTTACATGCATTATTA**<br>**GATAAATTAGCTAAGTCAGTAACATTATATCTTAATGCTCAAATTAA**<br>**GGCAGGTGCTCAAGCAGTTATGATTTTTGATACTTGGGGTGGAGTT**<br>**TTAACTGGAAGAGATTATCAACAATTTAGTCTTTATTATATGCATAA**<br>**AATTGTAGATGGATTATTAAGAGAAAATGATGGTAGAAGAGTACCA**<br>**GTAACTTTATTTACTAAGGGTGGTGGTCAATGGTTAGAAGCTATGG**<br>**CTGAAACAGGTTGTGATGCTCTTGGATTAGATTGGACTACTGATAT**<br>**TGCTGATGCTAGAAGAAGAGTTGGTAATAAGGTAGCACTTCAAGGA**<br>**AATATGGATCCAAGTATGCTTTATGCACCACCAGCTAGAATTGAAG**<br>**AAGAAGTTGCTACTATTCTTGCAGGATTTGGTCATGGAGAAGGTCA**<br>**TGTTTTTAATTTAGGACATGGAATACATCAAGATGTTCCACCAGAAC**<br>**ATGCTGGTGTATTTGTTGAAGCTGTTCATAGACTTTCAGAACAATAT**<br>**CATAGATAA**AAGGAGGTGTGTTACAT**ATGAGACAAACAAAGACAGGA**<br>**ATTCTTTTAGCAAATCTTGGTACTCCTGATGCACCAACACCTGAAGC**<br>**AGTTAAGAGATATTTAAAGCAATTTCTTAGTGATAGAAGAGTAGTT**<br>**GATACATCTAGACTTCTTTGGTGGCCTTTATTAAGAGGTGTTATTCT**<br>**TCCTCTTAGATCTCCAAGAGTTGCTAAATTATATGCTAGTGTTTGGA**<br>**TGGAAGGAGGATCACCTTTAATGGTTTATAGTAGACAACAACAACA**<br>**AGCACTTGCTCAAAGACTTCCAGAAATGCCAGTAGCTCTTGGAATG**<br>**AGTTATGGTAGTCCTTCATTAGAATCAGCAGTAGATGAATTATTAGC**<br>**TGAACATGTAGATCATATAGTTGTTTTACCATTATATCCTCAATTTT**<br>**CATGTAGTACAGTAGGAGCAGTATGGGATGAACTTGCTAGAATACT**<br>**TGCAAGAAAGAGATCAATTCCAGGTATTAGTTTTATTAGAGATTATG**<br>**CAGATAATCATGATTATATTAATGCATTAGCTAATTCTGTAAGAGCT**<br>**TCTTTTGCAAAACATGGTGAACCAGATTTATTACTTTTAAGTTATCA**

	**TGGTATTCCTCAAAGATATGCTGATGAAGGAGATGATTATCCACAA**<br>**AGATGTAGAACAACTACTAGAGAATTAGCATCAGCATTAGGTATGG**<br>**CTCCTGAAAAAGTTATGATGACATTTCAATCAAGATTTGGTAGAGA**<br>**ACCATGGCTTATGCCATATACAGATGAAACACTTAAAATGTTAGGA**<br>**GAAAAAGGTGTAGGTCATATTCAAGTAATGTGTCCAGGATTTGCAG**<br>**CAGATTGTTTAGAAACTCTTGAAGAAATAGCAGAACAAAATAGAGA**<br>**AGTTTTTCTTGGTGCAGGTGGAAAGAAGTATGAATATATACCAGCT**<br>**TTAAATGCTACTCCAGAACATATTGAAATGATGGCTAATTTAGTTGC**<br>**TGCATATAGATAA**AGACGGAGACG

Text S1. Supplementary methods

Plasmid construction (additional details for constructing other plasmids)

We constructed a series of vectors (number V1-1, V1-2, V1-3, V1-4 and V1-5 in) containing different targets and donor DNA templates based on the general vectors pP*fet*As-Target_v1 and pP*fet*Fn-Target_v1. For instance, vector numbered V1-3 was created from pP*fet*Fn-Target_v1 as follows: Firstly, pP*fet*Fn-Target_v1 was digested by AatII and SalI restriction enzymes (NEB), and the 5 kb fragment was purified as the backbone. Secondly, the fragments of the upstream and downstream regions for deleting the *spo0A* gene were amplified using the primer pair numbered 19/20, and 21/22 in Table S2, respectively, and fused by Golden Gate Assembly. Then, using the primer pair numbered 19/22, the donor DNA template fragment was amplified from the mixture of Golden Gate Assembly and digested by AatII and SalI restriction enzymes. Thirdly, the donor DNA template fragment and the backbone were ligated using T4 DNA ligase, resulting in vector pP*fet*Fn-D*spo0A*_v1, which was confirmed by Sanger sequencing. Finally, different target fragments were amplified as primer dimers and ligated with vector pP*fet*Fn-D*spo0A*_v1 using Golden Gate Assembly (BsmBIv2, NEB), resulting in vector numbered V1-3. Additionally, we constructed a series of vectors (V2-3, V2-4, and V2-5) based on the general vector pP*fet*Fn-Target_v2 in the same manner as described above.

To integrate a GusA expression cassette in *C. butyricum*, we constructed a series of vectors (number V2-6, V2-7, V2-8, V2-9, and V2-10) using the following steps: First, different target fragments were amplified and ligated to vector pP*fet*Fn-Target_v2 using the Golden Gate Assembly (BsmBIv2) to obtain vectors pP*fet*Fn-Inc.bGusA-Tn confirmed by Sanger sequencing. Second, vector pP*fet*Fn-Inc.bGusA-Tn was digested by AatII and SalI restriction enzymes, and the 6.5 kb fragment was purified as the backbone. Third, the upstream, GusA expression cassette, and downstream fragments were amplified using the primer pairs

numbered 103/104, 101/102, and 105/106 in Table S2, respectively, and fused by the Golden Gate Assembly. Donor DNA templates of different lengths were amplified from the Golden Gate Assembly mixture and digested with AatII and SalI restriction enzymes using primer pairs described in Table S2. Finally, the fragments and the backbone were ligated by T4 DNA ligase, resulting in vectors numbered V2-6, V2-7, V2-8, V2-9, and V2-10.

To integrate heterologous genes associated with fluorescence into the genomes of *C. butyricum* and *C. sporogenes*, the promoter fragment with Golden Gate sites in our previous work (1) (P*fdx*, P*thl*_fU, P*thl*79%-26_fU, miniP*c.b*_fU, miniP*4*_fU or miniP*c.sp*_fU) were ligated with gene fragment containing corresponding Golden Gate sites (IFP2.0-BsaI, UnaG-BsaI, CreiLOV-BsaI, HemO-BsaI, hemL-BsaI, RBS-hemA-BsaI, HemN-HemG-BsmBI or HemE-HemH-BsmBI) into vector pGG2121 by Golden Gate Assembly, obtaining the derivative vectors of pGG2121 (number P1, P2, P3, P4, P5, P6, P7, and P8). Then, we constructed a series of vectors (number V2-11, V2-12, V2-13, V2-14, V2-15, V2-16, V2-17, V2-18, and V2-19) to integrate gene expression cassettes. For example, vector numbered V2-11 was created as follows: We amplified the target fragment as the primer dimer and ligated it with the pP*fet*Fn-Target_v2 vector using Golden Gate Assembly (BsmBIv2) to create pP*fet*Fn-Inc.bIFP-T1, which was confirmed by Sanger sequencing. Next, we digested pP*fet*Fn-Inc.bIFP-T1 with AatII and SalI restriction enzymes and purified the 6.5 kb fragment as the backbone. Then, we amplified the upstream, IFP2.0 expression cassette and downstream fragments as described in Table S2 and fused them at Golden Gate Assembly. The donor DNA template fragment was amplified from the Golden Gate Assembly mixture and digested with AatII and SalI restriction enzymes. Finally, we ligated the donor DNA template and backbone fragments using T4 DNA ligase to obtain the vector numbered V2-11, containing the donor DNA template and target for

integrating the IFP2.0 expression cassette into *C. butyricum*, which was confirmed by Sanger sequencing.

Modified conjugation method

Both *E. coli* and clostridia were first grown separately overnight with the appropriate antibiotics. The ratio of *E. coli* donors to clostridia recipients was 5:1. Next, 1.5 mL of *E. coli* overnight culture (OD_{600} = 3.0) was centrifuged at 4000 ×g for 2 min, and the pellets were gently washed once in 1 mL of PBS. After heat treatment at 52°C for 5 min (3), 0.2 mL of clostridia overnight culture (OD_{600} = 4.5) was gently mixed with the *E. coli* pellets. The mixed culture was then placed on a Brain Heart Infusion (BHI) agar plate and incubated anaerobically for 8–9 hours at 37°C. Finally, the mating mixture was collected from the plate surface, suspended in 0.5 mL of PBS, and spread onto the appropriate plates.

Sporulation assay

Clostridium strains were cultured in PYT medium for ten days. The cells were then collected and suspended in the same amount of PBS. The heat resistance of colony-forming units (cfu) was evaluated by heating the samples at 80°C for 10 minutes, followed by serial dilution and plating.

Supplementary solutions

In this study, the supplementary solutions were prepared as follows: Chloramphenicol (C0378, Merck) was dissolved in ethanol to a concentration of 12.5 mg/mL; Erythromycin (E0751, TCI) dissolved in ethanol to a concentration of 30 mg/mL; D-Cycloserine (FC15208, BIOSYNTH) dissolved in water to a concentration of 50 mg/mL; Thiamphenicol (AT38721, BIOSYNTH) dissolved in 50% (v/v) ethanol to a concentration of 15 mg/ml; Anhydrotetracycline (37919, Sigma) dissolved in 50% (v/v) ethanol to a concentration of 32 μg/mL; D-glucose/Dextrose

(D9434, Sigma) dissolved in water to a concentration of 500 g/L; 5-FOA (R0811, Thermo Scientific) dissolved in DMSO to a concentration of 150 mg/mL; Bilirubin (14370, Merck), bliverdin (30891, Merck), riboflavin 5′-monophosphate sodium salt (F2253, Merck) and Hemin (51280, Sigma) dissolved in DMSO to a concentration of 25 mM; Menaquinone-4 (V9378, Sigma) dissolved in DMSO to a concentration of 12.5 g/L; Ferrous sulfate (PHR1483, Sigma) dissolved in diluted sulfuric acid to a concentration of 20 g/L.

References

1. Zhang Y, Bailey TS, Kubiak AM, Lambin P, Theys J. 2022. Heterologous Gene Regulation in Clostridia: Rationally Designed Gene Regulation for Industrial and Medical Applications. ACS Synth Biol 11:3817-3828.
2. Heap JT, Pennington OJ, Cartman ST, Minton NP. 2009. A modular system for *Clostridium* shuttle plasmids. J Microbiol Methods 78:79-85.
3. Kirk JA, Fagan RP. 2016. Heat shock increases conjugation efficiency in *Clostridium difficile*. Anaerobe 42:1-5.
